# Starch biosynthesis in cassava: a genome-based pathway reconstruction and its exploitation in data integration

**DOI:** 10.1186/1752-0509-7-75

**Published:** 2013-08-10

**Authors:** Treenut Saithong, Oratai Rongsirikul, Saowalak Kalapanulak, Porntip Chiewchankaset, Wanatsanan Siriwat, Supatcharee Netrphan, Malinee Suksangpanomrung, Asawin Meechai, Supapon Cheevadhanarak

**Affiliations:** 1 Bioinfromatics and Systems Biology Program, School of Bioresources and Technology, King Mongkut’s University of Technology Thonburi, 10150 Bangkok, Thailand; 2 Systems Biology and Bioinformatics Research Group (SBI), Pilot Plant Development and Training Institute, King Mongkut’s University of Technology Thonburi, 10150 Bangkok, Thailand; 3 Department of Chemical Engineering, King Mongkut’s University of Technology Thonburi, 10140 Bangkok, Thailand; 4 National Center for Genetic Engineering and Biotechnology, 10120 Pathumthani, Thailand; 5 Division of Biotechnology, School of Bioresources and Technology, King Mongkut’s Bangkok, Thailand

**Keywords:** Cassava, Data integration, Metabolic pathway reconstruction, Starch biosynthesis

## Abstract

**Background:**

Cassava is a well-known starchy root crop utilized for food, feed and biofuel production. However, the comprehension underlying the process of starch production in cassava is not yet available.

**Results:**

In this work, we exploited the recently released genome information and utilized the post-genomic approaches to reconstruct the metabolic pathway of starch biosynthesis in cassava using multiple plant templates. The quality of pathway reconstruction was assured by the employed parsimonious reconstruction framework and the collective validation steps. Our reconstructed pathway is presented in the form of an informative map, which describes all important information of the pathway, and an interactive map, which facilitates the integration of omics data into the metabolic pathway. Additionally, to demonstrate the advantage of the reconstructed pathways beyond just the schematic presentation, the pathway could be used for incorporating the gene expression data obtained from various developmental stages of cassava roots. Our results exhibited the distinct activities of the starch biosynthesis pathway in different stages of root development at the transcriptional level whereby the activity of the pathway is higher toward the development of mature storage roots.

**Conclusions:**

To expand its applications, the interactive map of the reconstructed starch biosynthesis pathway is available for download at the SBI group’s website (http://sbi.pdti.kmutt.ac.th/?page_id=33). This work is considered a big step in the quantitative modeling pipeline aiming to investigate the dynamic regulation of starch biosynthesis in cassava roots.

## Background

Cassava is an important food crop and ranked in the fourth place after rice, sugarcane and maize [1]. It has been reported to feed more than 700 million people per day [2]. Apart from the dietary aspect, cassava is also demanded by diverse industries, including medicine, cosmetics, biopolymers, and biofuels. These highlight the significance of cassava beyond its previous recognition as a staple food in many countries in the African and South American continents. Compared to other starchy crops, the competitiveness of cassava lies in the capacity for starch accumulation, and the tolerance to drought and the deprivation of soil nutrients; cassava roots contain starch up to 70 to 90 percent of the root dry weight [1] and cassava plants can grow under water scarcity and in low fertilized soil [3]. With the growing demand of starch along with a significant increase in world population, most of the research focuses on cassava still aim to produce cultivars with greater starch yield per unit cultivar area.

In contrast with the importance of cassava, a very limited amount of information on cassava is currently available in the literature. Before 2009, only a small number of the percentage of the cassava genome was revealed, mostly acquired from the classical genetic and EST expression studies [4]. Not only is there a lack of genome information, but the knowledge on cassava biology and physiology is also lacking and unorganized [5]. At the early stage, the genetic variability of cassava was explored and that information was utilized to breed for a novel cassava line [3]. Nowadays, various high-throughput technologies have been employed to disentangle the complex regulation underlying the biological processes in cassava plants, including cassava starch metabolism [2,6,7]. The availability of technologies to monitor the intracellular components, for example, the microarray and C-13 labeling experiment, has allowed researchers to investigate the regulation controlling the behavior of the cells. In 2009, with the release of the cassava genome sequence [8], the margin of the cassava research has leaped forward. The current sequenced genome of cassava covers 69 percent of the predicted genome size and contains 96 percent of the whole genic region. The 30,666 genes and 3,485 alternative splicing forms were predicted and annotated. This genome information has been made available at the Phytozome database [4]. The status of cassava genome information is much better after the sequencing, yet the real complete genome annotation is still on the way. In addition to the relatively little background knowledge on cassava in plant science research, an abundance of research questions about the cassava plant remain to be revealed, especially the regulation underlying the starch biosynthesis.

The current understanding of starch biosynthesis in cassava relies very much on the observations in the *Arabidopsis* model plant, and other starchy crops such as the potato. It has been believed that plant species share the backbone pathway of starch biosynthesis starting from the carbon dioxide fixation, followed by transitory starch degradation, sucrose synthesis, and starch synthesis in the storage organs. Sometimes, this conservation is also assumed at the level of biochemical reactions composed of the starch biosynthesis pathway. However, this presumption is questionable as to what would make the properties of plant starches diverse if the series of biochemical reactions comprised of the starch biosynthesis pathway in all plants are assumed identical. The discovery of the distinction between the starch biosynthesis pathways of cereal and non-cereal crops is the example evidence of the pathway diversity in plant species, highlighting the uniqueness of the specific pathway to the species. The major difference involves the conversion of glucose-6-phosphate (G6P) to ADP-glucose (ADPG), a common precursor for starch synthesis. In cereals, the conversion takes place in the cytosol and the ADPG is then imported to the amyloplast through a membrane-bound transporter, whilst in non-cereal crops, G6P is directly imported to the amyloplast prior to the G6P-to-ADPG conversion taking place [9]. Thus, to pursue a better understanding of starch biosynthesis in cassava, the species-specific pathway describing the series of reactions orchestrated in the network of the cassava starch production process is required.

The plant-specific metabolic pathways have continuously been reconstructed not only for the broad purposes as found in the public databases (*e.g.,* PMN [10] and KEGG [11]), but also for a particular pathway investigation as demonstrated in various publications (*e.g.,*[12]). Besides the information-rich *Arabidopsis*[13,14], the metabolic pathway reconstruction has been carried out in various valued crops, such as potato [15], wheat [16], barley [17] and maize [18]. For cassava, however, only broad metabolic pathways have been reported [6,10]. Though there are attempts to explore the starch biosynthesis in cassava using various techniques (*e.g.*, [8,19,20]), the most likely first metabolic pathways of cassava were credited to [6], whose pathways were inferred from the comparative genomic analysis of the full-length cDNA library and established before the release of the cassava genome data. Considering the starch biosynthesis pathway exemplified in their studies, it obviously showed an information deficiency containing several gaps in the resulting pathway. As a result, shortly after the availability of the cassava genome sequence in the Phytozome database, [21] revised such a pathway by using a more stringent comparative genomic protocol. The resulting pathway relatively contained more complete information, yet with further development, not only the quality of the reconstructed pathway would be improved, but the assurance for reliability and its further uses would also be confirmed.

Accordingly, we have improved the pathway reconstruction of starch biosynthesis in cassava by exploiting the cassava genome data and comparative genomic approach with multiple plant templates. This work was organized into two parts: pathway reconstruction and omics data integration. For the first part, the starch-related genes of cassava were identified through the sequence similarity analysis using five selected plant templates. The reconstructed pathway was verified by the cassava gene sequences from the GenBank database and the protein motif analysis, and then finally visualized in an informative and concise format. For the second part, the reconstructed pathway was transformed into the platform that facilitates the incorporation of high-throughput data into the network. Here, the microarray gene expression data was integrated into the reconstructed pathway of starch biosynthesis to illustrate the advantage of our resulting pathway to increase the comprehension of the studied system.

## Results and discussion

Given the importance of starch, biochemical processes underlying the starch biosynthesis pathway have been extensively studied in several plant species, but not in cassava. Therefore, this work aimed to explore the starch biosynthesis pathway in cassava using the comparative genomic approach. The resulting pathway of the starch biosynthesis in cassava is not only an innovation contributed from this study, but the observations along the reconstruction process are also useful to expand our comprehension on the studied process. The pathway reconstruction and the main observations are described as follows.

### The reconstructed metabolic pathway of cassava starch biosynthesis

**Figure 1:**
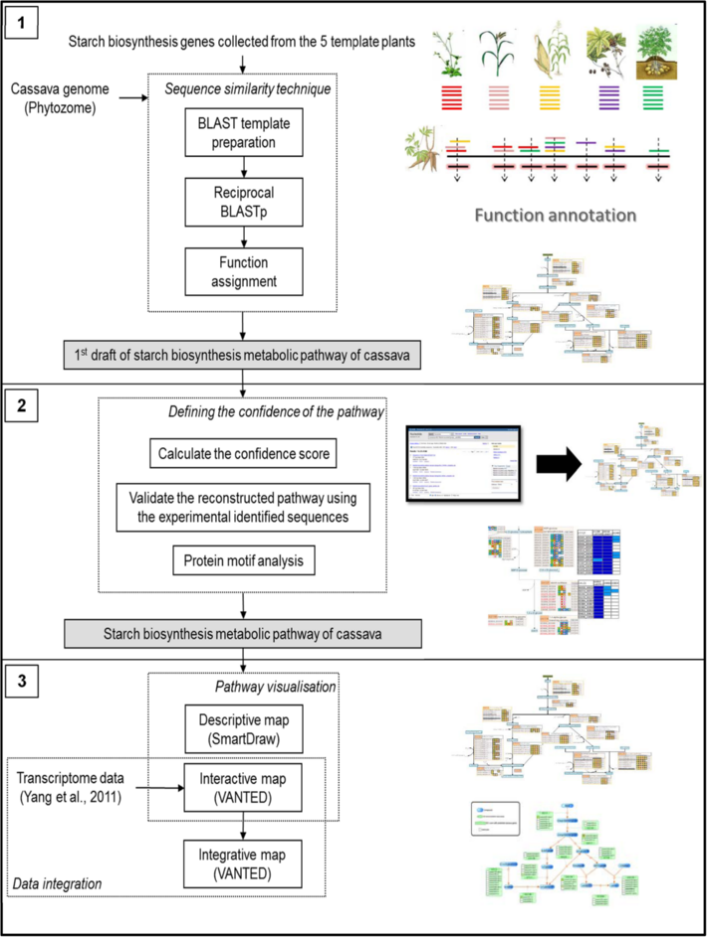
**The overview of the methodology.** The pathway reconstruction protocol is divided into three steps: **(**Panel **1)** finding the cassava orthologous sequences through the similarity search technique, **(**Panel **2)** defining the confidence of the cassava sequence annotation as well as the pathway reconstruction, and **(**Panel **3)** visualizing the resulting pathway.

The starch biosynthesis pathway, which covers three main processes including the Calvin cycle, sucrose synthesis, and storage starch biosynthesis, was reconstructed in this work. The reconstruction protocol was developed based on the similar basic idea as [21] in the exploitation of the multiple template plants for the pathway annotation, but it was re-designed to incorporate certain intensive analyses to ensure the high-level of quality of the resulting pathway. As outlined in Figure 1, multiple plant species were employed to identify the set of starch-related genes in cassava. After a manual pathway curation, the resulting pathway was further validated to certify the accuracy of the reconstruction. Furthermore, the confidence scoring system and informative as well as interactive visualization maps were created to increase the value of the reconstructed pathway. The results from each analysis are discussed as follows.

**Table 1:** The number of cassava protein sequences annotated to be involved in the starch biosynthesis pathway

**Template plants**	**Sucrose and starch synthesis**		**Calvin cycle**	
	**No. of template sequences**	**No. of cassava sequences annotated**	**No. of template sequences**	**No. of cassava sequence annotated**
*Arabidopsis*	103	110	79	115
Maize	198	117	125	74
Rice	121	118	116	124
Castor bean	51	79	51	101
Potato	130	96	109	109
Total		**141**		**129**

The starch biosynthesis pathway of cassava here is comprised of 270 cassava protein sequences annotated by their orthologues in the five template plants, *Arabidopsis*, maize, rice, castor bean and potato. Among the annotated sequences, 141 proteins were found to be involved in the sucrose and starch synthesis sub-pathway and the remaining 129 proteins were related to the Calvin cycle sub-pathway.

**Figure 2:**
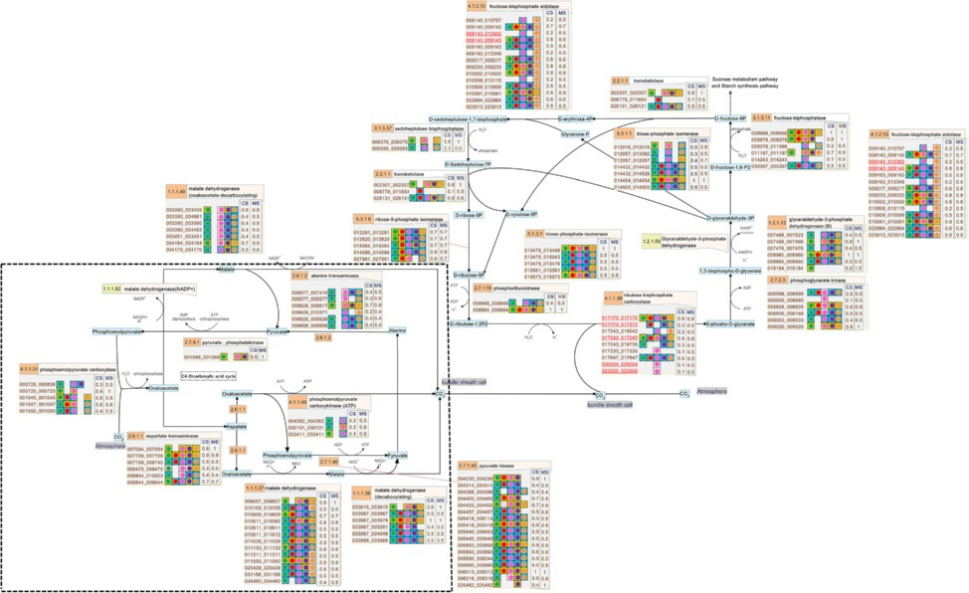
**The reconstructed pathway of the carbon dioxide fixation process in cassava presented on the SmartDraw platform.** The number in the orange boxes denotes the EC number of the enzymes which is possibly a product of the genes below, denoted as the 12-digit ID. The colored dots beside each gene ID indicate the plant templates from which the genes were annotated: green – *Arabidopsis*, red – maize, pink – rice, violet – castor bean, and orange – potato. The background colors of the dots represent the matching quality of the sequence alignment: highest in yellow to lower in blue and the lowest in white. The following two columns describe the match (*MS*) and conservation (*CS*) scores, respectively. The box marks the reactions that are relevant to the C4-photosynthesis pathway found in a typical C4-plant.

**Figure 3:**
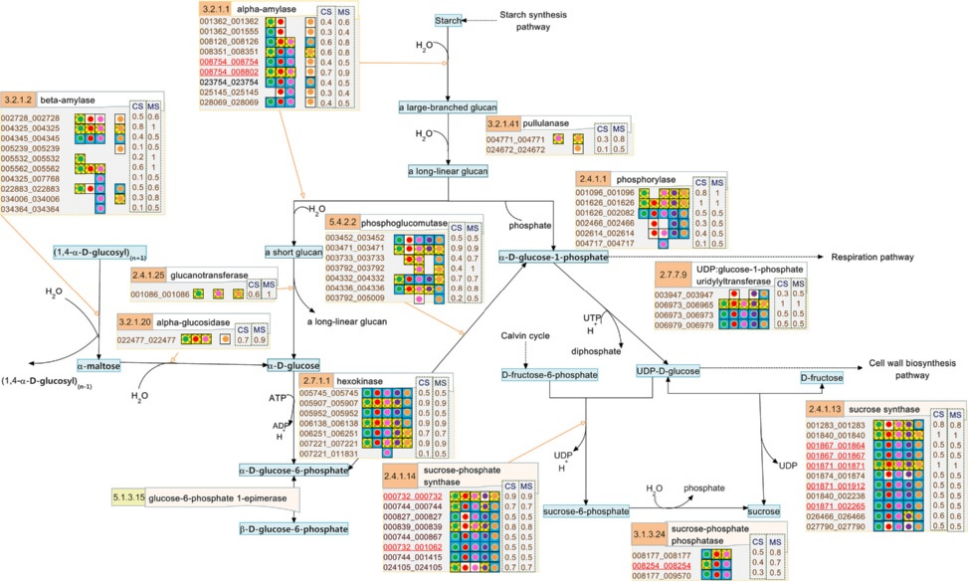
**The reconstructed pathway of the sucrose synthesis process in cassava presented on the SmartDraw platform.** The number in the orange boxes denotes the EC number of the enzymes which is possibly a product of the genes below, denoted as the 12-digit ID. The colored dots beside each gene ID indicate the plant templates from which the genes were annotated: green – *Arabidopsis*, red – maize, pink – rice, violet – castor bean, and orange – potato. The background colors of the dots represent the matching quality of the sequence alignment: highest in yellow to lower in blue and the lowest in white. The following two columns describe the match (*MS*) and conservation (*CS*) scores, respectively.

**Figure 4:**
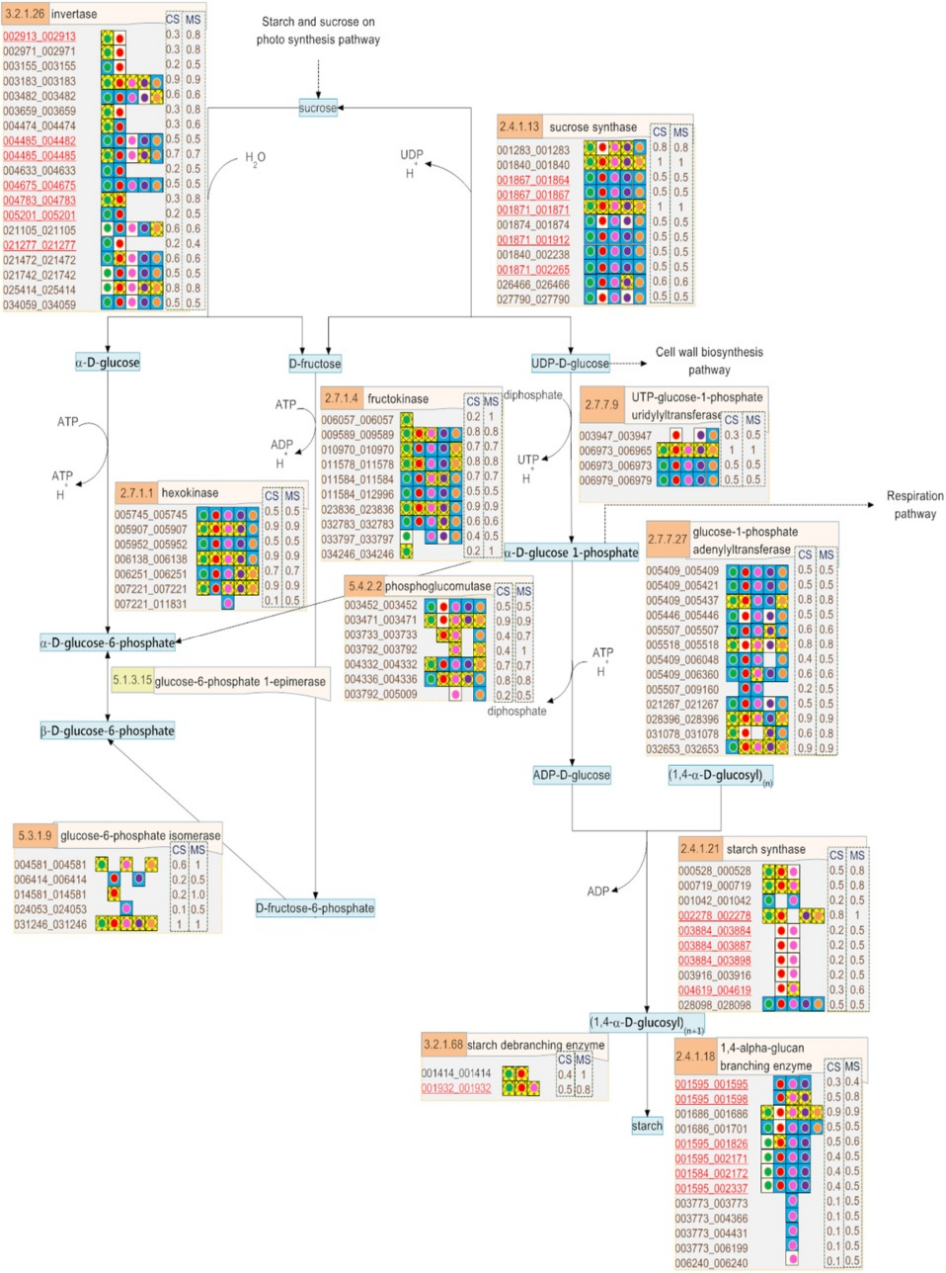
**The reconstructed pathway of the starch synthesis process in cassava presented on the SmartDraw platform.** The number in the orange boxes denotes the EC number of the enzymes which is possibly a product of the genes below, denoted as the 12-digit ID. The colored dots beside each gene ID indicate the plant templates from which the genes were annotated: green – Arabidopsis, red – maize, pink – rice, violet – castor bean, and orange – potato. The background colors of the dots represent the matching quality of the sequence alignment: highest in yellow to lower in blue and the lowest in white. The following two columns describe the match (MS) and conservation (CS) scores, respectively.

According to sequence similarity, 270 starch-related proteins in cassava (see more detail in Additional file 1) were identified from their orthologues collected from the five template plants, including *Arabidopsis*, rice, maize, castor bean and potato. Among these, 141 proteins were observed to play a role in the sucrose and starch metabolism, while the remaining 129 proteins have functions in the Calvin cycle (Table 1). According to the KEGG and PMN reference pathway databases, our reconstructed pathway of starch biosynthesis comprised of the identified proteins was broken up into three aforementioned sub-pathways and then presented on both SmartDraw (Figures 2, 3, 4) and VANTED (Additional file 2: Figure S1; Additional file 3: Figure S2; Additional file 4: Figure S3) platforms. Due to the lack of a metabolic gap, we are claiming that the starch biosynthesis pathway of cassava was successfully reconstructed from the 270 cassava proteins identified by these five template plants. Compared to the two previous pathways proposed by Sakurai *et al.* (2007) and Rongsirikul *et al.* (2010), our reconstructed pathway definitely provides more complete information on the process underlying the starch biosynthesis in cassava [6,21].

The descriptive map drawn on the SmartDraw platform was mainly used to explain the nature of the starch biosynthesis pathway in cassava (see more detail in Section “Inferring the nature of the cassava starch biosynthesis pathway through comparative genomic study” of Results and discussion) because it included the information from which the pathway was reconstructed. In addition to a set of biochemical reactions and a number of involving metabolites, our descriptive map also includes the protein lists that were identified to play roles in specific reactions according to a given EC number (Figures 2, 3, 4). For a protein in the list, there is a graphical notation indicating (i) the organisms from which the protein was annotated and (ii) the confidence level of the function assignment. The descriptive maps that appeared on the SmartDraw platform utilized (i) colored circles to represent the plant species: green – *Arabidopsis*, red – maize, pink – rice, violet – castor bean, and orange – potato. They also utilized (ii) colored boxes to represent the confidence level of the function assigned: yellow > blue > white. Furthermore, the sophisticated confidence scores that numerically quantify the reliability of the protein annotation were also included in this map. All proteins presented in the map were given a 12-digit numeral ID whereby the first 6 digits represent the gene ID as available on the Phytozome database and the rest is the transcript ID of the corresponding genes. This system allows traceability between the reconstructed map and the reference genome database.

**Figure 5:**
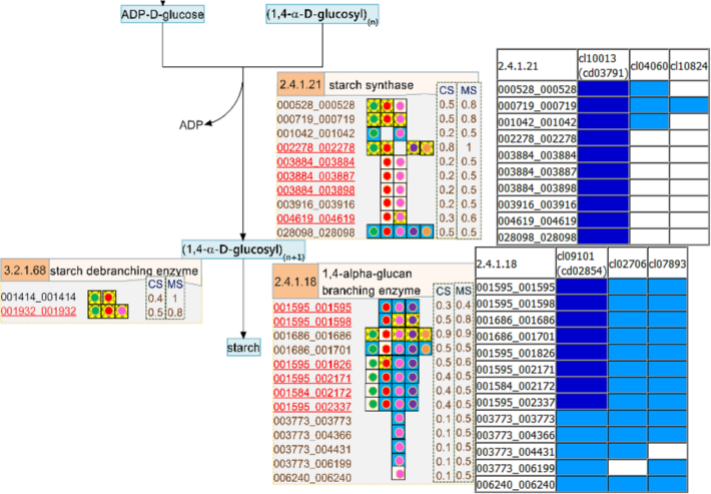
**The example of the pathway map that includes the results of the protein motif analysis.** The functional motifs of the proteins were examined through the Conserved Domain Database (CDD) web tool from the NCBI database (http://www.ncbi.nlm.nih.gov/Structure/bwrpsb/bwrpsb.cgi). The protein motifs contained in the analyzed sequences are presented in two levels: specific hit domain (dark blue - indicates a highly confident annotation fit to a specific motif in the NCBI repository) and superfamily (light blue - indicates a collection of the motifs that are found redundantly among the homologous proteins). See the detailed definition and types of identified motifs in [41,42].

Besides the completeness of the resulting pathway and the informative visualization maps, the quality of the reconstructed pathway was increased by introducing manual curation and literature-based validation into the reconstruction framework. After human curation, the accuracy of the pathway was proven through two tests. The first test intended to demonstrate the correspondence between the reconstructed pathway and the existing data of cassava published in literature. The hypothesis underlying this test is that the well-reconstructed pathway should be able to match the experimentally identified genes of cassava existing in the GenBank database. The positive results were obtained as it showed that 18 starch-related genes of cassava (corresponding to 36 identified proteins in our pathway) matched with the annotated genes in our starch biosynthesis pathway, marked as underlines in Figures 2, 3, 4. Although the results of the first test suggest a good quality of the reconstructed pathway, it is important to note that this suggestion was inferred from the validation of only 13% (36/270) of all annotated genes. To provide further evidence supporting the correctness of our pathway, the second test was carried out to investigate the correspondence between the annotated function and the conserved motifs existing in the protein. For example, the protein that was annotated as amylase was analyzed to find whether it truly contains the catalytic motifs highly conserved for all amylases. The results of this test confirm the validity of the protein annotation as well as the pathway reconstruction herein. Figure 5 exemplifies a part of the interactive map where the results of the analysis were demonstrated. The complete results are provided as the supplementary data (Additional file 5) or can be downloaded from the website of the Systems Biology and Bioinformatics research group at KMUTT (http://sbi.pdti.kmutt.ac.th/?page_id=33).

Taking all results together, our study offers the newly reconstructed pathway of starch biosynthesis in cassava with greater quality over that presented in the literature. The quality of the pathway here is not only defined by the parsimonious reconstruction framework, but also the visualization styles enhancing the pathway utilization for further research. With respect to the most recently proposed pathway of cassava starch biosynthesis [21], which contained less confident annotation with weak validation process, the contribution of this work provides a higher-quality pathway of starch biosynthesis in cassava.

### Inferring the confidence of the gene annotation through confidence scores

**Figure 6:**
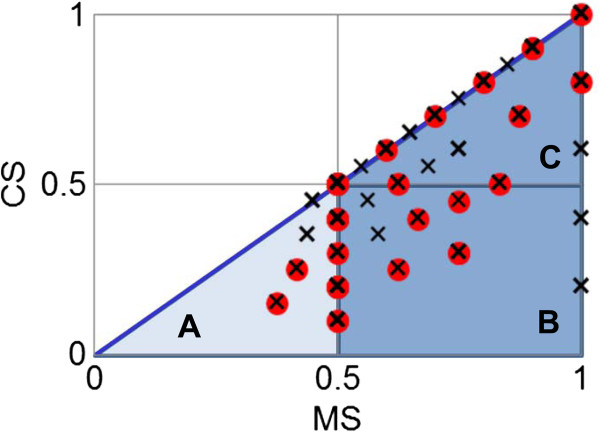
**The scheme plotting the relationship between the match score (*MS*) and conservation score (*CS*).** The shadow region refers to the theoretical range of both confidence scores (*MS* and *CS*) and the circles indicate the scores of sequences whose identification was supported by the experiment. The regions **A**, **B**, and **C** refer to the area in the *MS-CS* plot having different levels of confidence: **A** – low, **B** – high, and **C** – extremely high.

Scoring is a strategic approach to project the abstract quantity onto the measurable standard, so the confidence scores, *i.e., MS* and *CS*, are meant to numerally measure the reliability of the protein annotation constituting the reconstructed starch biosynthesis pathway. The advantages of these quantitative scores can be described in three levels as they were employed in this work. The basic advantage lies on the straightforward use of the scores to represent the level of confidence of each protein annotation (Figures 2, 3, 4). At the higher level, the scores were exploited to make a clear comparison between the annotation qualities among the annotated proteins. It enabled us to rationally prioritize the proteins for further investigation. For the advanced advantage, the scores were utilized on the purpose of inference and prediction. In this context, we created the *MS-CS* plot where the *MS* scores of all annotated proteins were plotted against their corresponding *CS* scores to investigate the distribution of these scores in the overall range (Figure 6). Each cross mark represents the *MS-CS* scores of a protein member in the starch biosynthesis pathway and the circles highlight the scores of proteins that were certified by the GenBank database to exist in cassava with corresponding annotation to our reconstruction. Figure 6 shows that the *MS-CS* plot can be divided into three regions according to confidence levels: A (low confidence) – poorly matched with the template sequence in alignment analysis (low *MS*) and poorly conserved across plant templates (low *CS*), B (high confidence) – well matched with the template sequence in alignment analysis (high *MS*) but poorly conserved across plant templates (low *CS*), and C (extremely high confidence) – well matched with the template sequence in alignment analysis (high *MS*) and highly conserved across plant templates (high *CS*). As demonstrated, almost all of the annotated proteins supported by the data in GenBank (~ 90%) possess the confidence scores in regions B and C, so that it might be deduced that the proteins whose confidence scores fall into regions B and C were considered as the confident annotation and worthwhile of future analysis.

### Inferring the nature of the cassava starch biosynthesis pathway through comparative genomic study

In this study, the cassava starch biosynthesis pathway was reconstructed from information retrieved from multiple template plants. With this method, we have discovered interesting information regarding the nature of the starch biosynthesis pathway in the plants under study, including the conservation and the uniqueness of the process.

The conservation of the starch biosynthesis process among the six plant species, including cassava and the five template plants, were observed at both molecular sequence and pathway levels (Figures 2, 3, 4). In analogy to the orthologous or conserved gene identification from the similarity of sequences, the conservation at the pathway level may be inferred from the similarity of the constituents and the structure of the pathway. It was shown in our results that the molecular sequences comprised of the starch biosynthesis pathway were highly conserved in plant species, though their starch-storage organs are physiologically different. For example, the genes encoding for sucrose synthase (EC 2.4.1.13) and ADP-glucose pyrophosphorylase (EC 2.7.7.27), the main components of the starch biosynthesis pathway [22,23], were found in nearly all plant species; moreover, these orthologous sequences show a high degree of sequence likeliness, at least with the sequence of cassava. The observed conservation of the important enzymatic proteins in the starch biosynthesis pathway of cassava is not only limited to the class of dicotyledonous plants (*i.e.,* Arabidopsis, potato and castor bean), but also found across the monocotyledonous plants (*i.e.,* rice and maize; Figure 4). The conclusive view of this observation was demonstrated as the high confidence scores in the *MS-CS* plot (region C) to which the resemblance of the aligned sequences and the frequency of finding such sequences in the list of studied species were referred, respectively (Figure 6). At the pathway level, we showed that the starch biosynthesis pathway of cassava is very similar to the pathway of the other plants established in the KEGG and PMN reference databases (KEGG: http://www.genome.jp/kegg/; PMN: http://www.plantcyc.org/) in terms of the number of biochemical reactions and enzymatic proteins. Almost all of the enzymatic proteins of the starch biosynthesis existing in the reference pathway, which collects information from various plant species including rice and Arabidopsis model plants, were also found to be encoded in the cassava genome and presumably formed a similar shape to the metabolic pathway as illustrated in Figures 2, 3, 4. The apparent conservation or the similarity of the starch biosynthesis pathway among plants indicates their common ability to synthesize starch in cells. However, at this level of detailed pathway reconstruction, it might be too early to infer the complete picture of the intracellular starch production process, covering the exact mechanism of starch granule formation. This downstream part of the synthesis pathway remains mysterious, yet the related presumptions and findings have been found in various reviews [24-27]. Regarding the high similarity of the core biochemical pathway of the starch biosynthesis, it might suggest that the factors dictating the variation in plant starches, in terms of characteristics and accumulation capacity in the cells, might be hidden in the granule formation step where the enzymes (also isozymes) work together in a very precise order [23,27,28]. Another explanation of the distinct starch production yielding of the conserved pathway might be the dissimilarity of enzyme kinetics and the dynamics of the process, which are supposed to be a species-specific property. The implication of this hypothesis lies on the findings of the distinct activity and specificity of the same enzyme in organisms as well as plant species [22,25,29]; an example includes varied activities of starch branching enzyme isoforms between dicotyledon and monocotyledon plants (reviewed in [28]).

Despite the high conservation of the starch biosynthesis pathway, the results indicated the variation of genes responsible for the starch biosynthesis process in a particular plant, implying the species-dependent pathway uniqueness. As observed in Figures 2, 3, 4, some of the genes could be identified in only one or two template plant species. These include the genes that code for starch synthases (EC 2.4.1.21) (*e.g.*, 001042_001042, 003884_xxxxxx, and 004619_004619), the 1,4-alpha glucan branching enzyme (EC 2.4.1.18) (*i.e.,* 003773_xxxxxx), and the starch debranching enzyme (EC 3.2.1.68) (*i.e.,* 01414_001414). This type of information was found more often in the sucrose and starch biosynthesis pathway, but not in the Calvin cycle. Our interpretation was reinforced, not an exaggerated conclusion from the low confident data, by the observation that the two (*i.e.,* 003884_xxxxxx, and 004619_004619) of three experimentally supported genes related to starch synthase enzyme were identified from only two template plants (maize and rice). Overall, the identified genes falling into region B of the *MS-CS* plot, possessing low *CS* but high *MS* scores, reflect the uniqueness of the starch biosynthesis pathway in the cassava. To address the stronger hypothesis of which uniqueness results in the distinction of cassava starch biosynthesis from the others, more investigation is required.

Another interesting observation is the long list of genes/proteins predicted to be involved in a single metabolic reaction in the starch biosynthesis pathway (Figures 2, 3, 4). On average, approximately five, four, and seven cassava genes were predicted to be participants in one metabolic reaction in the Calvin cycle, sucrose synthesis and storage starch biosynthesis, respectively. These identified genes/proteins in the list, moreover, show high conservation across plant species, suggesting that the results are not false positives, but instead reflect the complexity of the starch production process in cassava. The complexity of the metabolic process due to the multiple proteins modulating a single reaction often refers to the functional redundancy and mutual participation scenarios. The circumstance supporting the existence of these scenarios in plants would be the presence of multiple isoforms of starch-related enzymes, *e.g.,* starch synthase and starch branching enzymes, for converting one metabolite to another (Additional file 6: Figure S4; Additional file 7: Figure S5; Additional file 8: Figure S6). Also, it has been found in the range of plant species that these enzyme isoforms contain redundant functions to each other and sometimes mutually participate in glucosyl chain extensions in plants [30-32].

**Figure 7:**
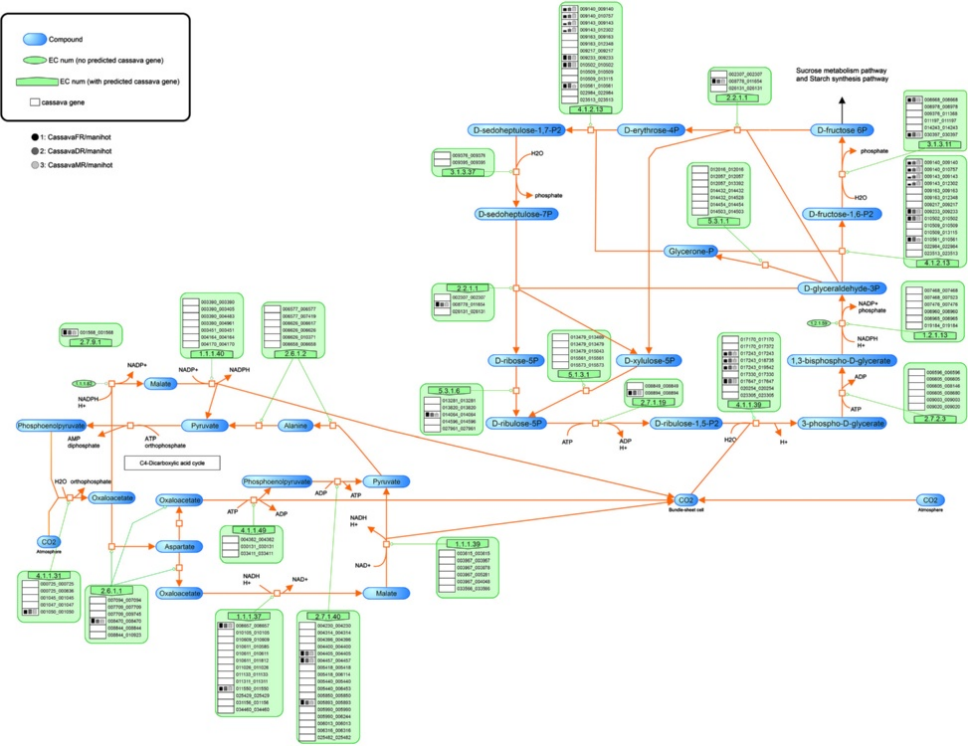
**The integrated map of the carbon dioxide fixation process presented on the VANTED platform.** The microarray data measuring gene expression in fibrous, developmental and mature roots of cassava were integrated into the reconstructed pathway. The height of the bar graph indicates the level of gene expression, while the colors denote the types of roots: black - fibrous root, dark grey - developmental root, and light grey - mature root.

At last, we presented results that could be additional evidence supporting the classification of cassava as a C3-C4 intermediate species. Photosynthesis in cassava has been researched since the 1970s. With continuous research, the classification of cassava has changed dramatically from being a C3 plant [33] to that with C4 photosynthesis characteristics based on physiological and biochemical studies [34,35], and nowadays to a C3-C4 intermediate plant [35-37]. In this study, the findings of C4-phosynthesis-related genes in the cassava genome (see the reconstructed pathway in Figure 2) and their transcriptional activities (see integrated map; Figure 7) reinforce the possession of C4-phosynthesis characteristics in cassava. The metabolic pathway reconstruction of CO_2_ fixation (Figure 2) predicted that several genes were responsible for the C4-photosynthesis pathway (marked as box in Figure 2), including the genes encoding phosphoenolpyruvate carboxylase (PEP Case; EC 4.1.1.31) which is a key enzyme of the C4-photosynthesis pathway. The activities of these cassava genes were obviously observed at the transcriptional level (Figure 7; more discussion in the next section); however, it has been well established that their activities at the metabolic level are low with respect to that of the typical C4-plants [35,37].

### Insight into starch biosynthesis pathway/regulation through data integration

**Figure 8:**
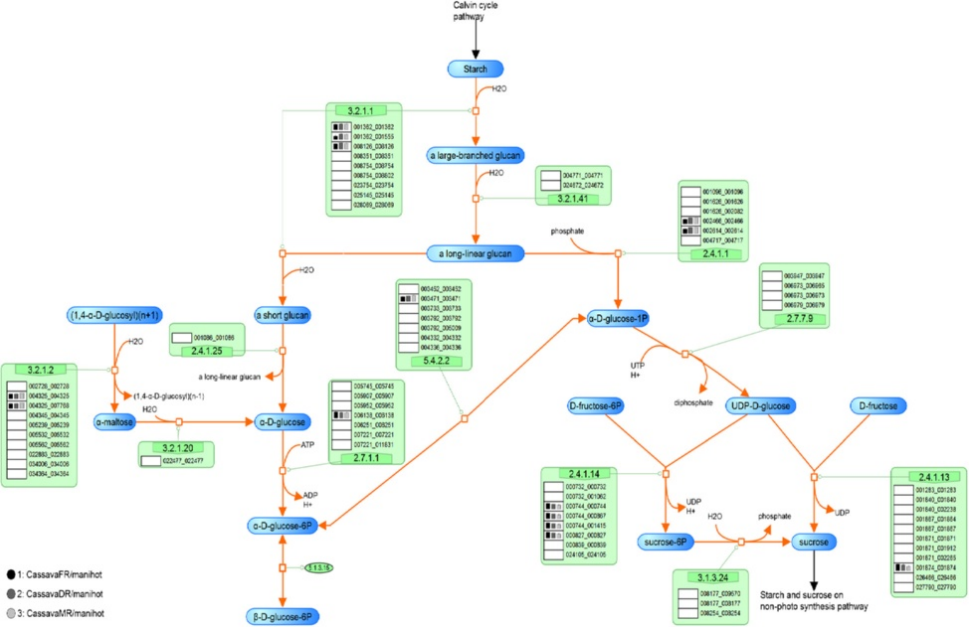
**The integrated map of the sucrose synthesis process presented on the VANTED platform.** The microarray data measuring gene expression in fibrous, developmental and mature roots of cassava were integrated into the reconstructed pathway. The height of the bar graph indicates the level of gene expression, while the colors denote the types of roots: black - fibrous root, dark grey - developmental root, and light grey - mature root.

**Figure 9:**
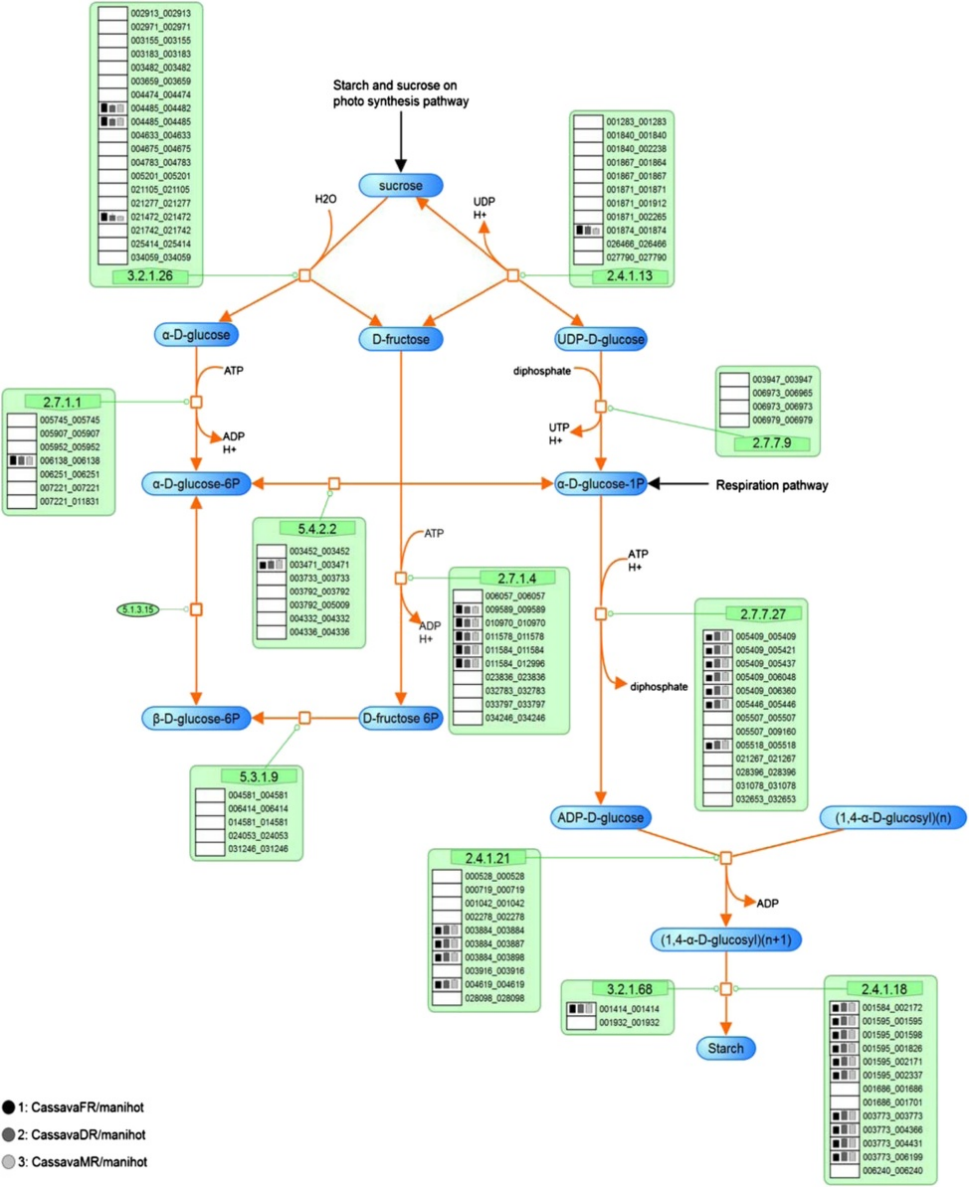
**The integrated map of the starch synthesis process presented on the VANTED platform.** The microarray data measuring gene expression in fibrous, developmental and mature roots of cassava were integrated into the reconstructed pathway. The height of the bar graph indicates the level of gene expression, while the colors denote the types of roots: black - fibrous root, dark grey - developmental root, and light grey - mature root.

In this section, the reconstructed pathway was used as a platform for omics data integration to enhance our comprehension on the starch biosynthesis in cassava. This strategy enables us to gain more understanding into the process by observing the interrelation between the multilevel regulations. To illustrate the benefit of our approach and the innovated pathway, the microarray data of [7] were integrated into the cassava starch biosynthesis pathway in the VANTED platform (Additional file 2: Figure S1; Additional file 3: Figure S2; Additional file 4: Figure S3). In brief, this microarray experiment measured changes in gene expression patterns in three different types of cassava roots, including fibrous, developing storage and mature storage roots which are believed to possess distinct predominant metabolism, especially those related to starch biosynthesis. Figures 7, 8, 9 depict the results after incorporation of the microarray gene expression data. The height of bars represents the gene expression levels and the color of each bar represents the types of roots being studied: the fibrous root – is black, the developing storage root – is dark grey, and the mature storage root – is light grey. According to the integrative map (Figures 7, 8, 9), our findings based on the inter-relationship between the transcriptomic and metabolic level of regulation can be drawn as follows.

According to Figure 7, the expression of genes commonly known to play a role in the CO_2_ fixation process was also observed in cassava roots. The observation of the activity of the photosynthesis-related genes in the roots of cassava is not entirely unusual because there are similar findings in other green plants like *Acmella*, *Artemisia*, *Rudbeckia*, *Sfevia*, *Tagetes* and orchid roots [38-40]. The literature, moreover, suggested that the CO_2_ fixation in roots or underground organs may take place via the PEP Case enzyme rather than Ribulose-1,5-bisphosphate carboxylase (RuBP Case; EC 4.1.1.39) [40]. Regarding the observed gene expression of the PEP Case in cassava roots, it is possible that the PEP Case enzyme retains its function as it does in green root plants, and thus CO_2_ might be fixed into cassava roots via the same mechanism.

When comparing the expression of genes in the CO_2_ fixation pathway between the three different root types, the results suggested that the Calvin cycle is more active over the C4 pathway. This interpretation was drawn from the observation that the expression of genes in the Calvin cycle evolves with the root types, while the C4 pathway is tentatively independent of the types of cassava roots. Our results correspond to the finding that the activities of enzymes in the C4 pathway are less dominant in cassava CO_2_ fixation with respect to the activities of key enzymes in the Calvin cycle [3,35]. However, the demonstrated gene expression indicated the activity of the C4 photosynthesis pathway in cassava, at least at the transcriptional level.

In the Calvin cycle, the genes required for the synthesis of triose sugars (3-phosphoglyceric acid, 3PGA and glyceraldehyde 3-phosphate, PGAL) are highly expressed in the mature storage roots over their expression in the developing storage and fibrous roots (Figure 7). The results might reflect the increasing demand of the precursors for starch biosynthesis and cellular activities in the mature root where starch is highly produced and accumulated compared to the other root types.

For sucrose and starch biosynthesis pathways (Figures 8 and 9), the integrated map suggested a sensible illustration of the progressive starch production along with the cassava root development. The expression of genes encoding the enzymes that catalyze the reactions toward starch production is highly expressed in the mature storage roots with respect to that in the fibrous and developing storage roots, indicting the dominance of this metabolic process in the storage root type. These results might be unsurprising and simply be forecasted from the existing knowledge of starch biosynthesis. However, it is worthwhile to investigate and confirm this circumstance to ensure the current understanding.

## Conclusions

Nowadays, the importance of cassava is unquestionable both in terms of being a significant food stock and a promising energy source. Since the advantage of cassava over the other crops lies in the incredible amount of starch produced and accumulated in the underground roots, we paid our attention to the starch biosynthesis process in the cassava roots. Employing the recently released cassava genome data, this study presented the high-quality pathway of cassava starch biosynthesis reconstructed using multiple plant templates. The quality of the pathway was claimed as including curation as well as validation steps into the reconstruction framework, *i.e.,* the curated pathway was verified by the information from the GenBank database and also by the protein motif analysis. Moreover, we have increased the distinction of our reconstructed pathway by numerally indicating the level of confidence for each sequence annotated here and the manifold visualizations. The confidence scores are considered advantageous in two aspects: (1) to quantitatively indicate the confidence level of the comparative results and (2) to suggest the reliability level of the newly annotated genes from the computational prediction. This information could be a hint for a molecular biologist to select the genes of interest for further investigation. For the pathway visualization, in addition to the descriptive view of the pathway that is often deduced from all pathway reconstruction research, we have also provided the resulting pathway in the interactive form. This format of visualization facilitates omics data integration (*e.g.,* transcriptome data from the microarray experiment) to allow the researchers to easily outline a new hypothesis regarding the regulation of the starch biosynthesis process. At the end, we exemplified the usefulness of the reconstructed pathway by incorporating the gene expression data [7] to understand the nature of starch biosynthesis in the cassava plant. The success of this work inspired us to extend the reconstruction to other starch-relevant pathways. In the near future, it might be possible to have a quantitative model to observe the dynamic of the regulation of starch biosynthesis in cassava. If the conservation of the regulation underlying behavior of the root crop species was proven, the understanding from cassava research would be of great benefit for other plant research. In this case, cassava, whose potential in terms of data availability is dominant over the starchy-root crops, could be considered as a model of root crop species. Therefore, the current effort of cassava study would be a real contribution for the research on other starchy root plants in the future.

## Methods

This work aimed to investigate the starch biosynthesis process in cassava through metabolic pathway reconstruction and data integration. The work was, therefore, divided into two parts: the reconstruction of the high-quality starch biosynthesis pathway and the exploitation of the reconstructed pathway to enhance the understanding on such a process via omics data integration. The complete workflow of this study is exhibited in Figure 1. The top two panels (labeled as 1 and 2) describe the two steps comprised of the pathway reconstruction part, the comparative-based method of pathway reconstruction and the validation protocol; the bottom panel illustrates the pathway visualization and data integration consisting in the second part.

### Pathway reconstruction through the sequence similarity search technique using multiple plant templates

Since the cassava genome sequence has not yet been fully annotated, an attempt to identify the starch-related genes in cassava was carried out using the inverse-comparative genomic approach, whereby the genes of interest in cassava were identified by the set of their orthologues in the template plants. Basically, instead of performing the cassava genome annotation to identify the genes of interest, we utilized the information derived from multiple well-studied plants to search for the gene orthologues in cassava.

#### BLAST template preparation

Five plants, consisting of *Arabidopsis thaliana*, rice (*Oryza sativa*), maize (*Zea may*), castor bean (*Ricinus communis*), and potato (*Solanum tuberosum*), were used as templates based upon any of the following criteria: having well-defined genome information, being a starch crop, and being evolutionarily or physiologically close to cassava. To provide information related to the starch biosynthesis pathway, the metabolic processes to be covered in this study would start from those involved in the fixation of atmospheric carbon atoms to the production of storage starch in roots. For template plants, the genes in these processes were identified based on the information available in the KEGG (Kyoto Encyclopedia of Genes and Genomes, *i.e.,* carbon fixation in photosynthetic organisms (map00710) and the starch and sucrose metabolism pathway (map00500)) and PMN (Plant Metabolic Network, *i.e.,* Calvin-Benson-Bassham cycle, sucrose and starch metabolism I and II, sucrose biosynthesis, sucrose biosynthesis II, starch degradation I and II, and starch biosynthesis) pathway databases. Additionally, the protein sequences of each template plant were retrieved from the following genome databases: *Arabidopsis* – TAIR database (http://www.arabidopsis.org/), rice – rice genome annotation project (http://rice.plantbiology.msu.edu/), maize – Gramene database (http://www.gramene.org/Zea_mays/Info/Index), castor bean – KEGG database (http://www.genome.jp/kegg/), and potato – Solgenomic database (http://solgenomics.net/). For cassava, the genome information was acquired from the Phytozome database version 4.1 (http://www.phytozome.net/; Phytozome 7 and cassava genome version 4.1). Table 1 summarizes the number of proteins involved in the starch biosynthesis in each template plant collected from both KEGG and PMN databases. This information was utilized as template queries in the next sequence alignment analysis to identify their orthologues in cassava.

#### Reciprocal BLASTp

Reciprocal BLAST is the bidirectional sequence alignment which is usually employed in the computational prediction of the putative orthologue in an unknown organism. All sequence alignments were performed through stand-alone BLASTp version 2.2.23. Firstly, we utilized a particular protein sequence derived from the template plant as a query to compare against the cassava protein library, denoted as the first BLASTp. Subsequently, the cassava proteins derived from the first BLASTp were then used to align against the protein library of the template plant, denoted as the second BLASTp. The critical values of the alignment were set as follows: E-value ≤ 1e-10, identity percentage (indicates the similarity between the aligned sequences with respect to the length of the matched region) ≥ 60, and coverage percentage (indicates the similarity between the aligned sequences with respect to the size of the query’s sequence) ≥ 80. Only the cassava sequences that possess the BLASTp scores above the set critical values would be further analysed.

#### Function assignment

The function of cassava protein sequences were assigned in accordance with their orthologues in template plants. Two types of identified cassava protein sequences were given the function herein. One is the best hit of the first BLASTp, thus potentially representing a reciprocal best hit (RBH) sequence, and the others are the rest of the resulting sequences whose scores are over the threshold in the first BLASTp. The function of the RBH sequences were always assigned based on the function of the template sequence, whilst the non-RBH sequences were given the function only if their best match in the second BLASTp had an identical function to that of the template. The cassava proteins annotated from these two methods were given unequal confidence scores (see the Section of “Confidence score calculation”), allowing the distinguishability between the types of prediction in the visualized pathway map.

### Validation and confidence evaluation of the pathway

#### Pathway validation

The reconstructed pathway was literately verified through two processes. Firstly, the reconstructed pathway was proven to correspond to the experimental data publicly available in the GenBank database. In practice, the pathway was tested to determine whether it included the starch-related genes in cassava by using the resulting pathway as a template to search for all starch-related genes in cassava whose sequences are available in the GenBank database. Additional file 9: Table S1 lists 18 cassava genes that could be retrieved and aligned with respect to the annotated cassava sequences in our pathway (E-value ≤ 10^-10^ and percent identity ≥ 90). Secondly, the plausibility of the cassava protein annotation was consolidated by the existence of the conserved motifs related to the function given to the protein. The conserved protein motifs were identified by using the Batch CD-Search, a Conserved Domain Database (CDD) web tool from the NCBI database (http://www.ncbi.nlm.nih.gov/Structure/bwrpsb/bwrpsb.cgi; [41,42]). The identified conserved motifs in a protein sequence were presented in two classes: specific hit domain, the highly confident annotation fit to a specific motif in the NCBI repository, and superfamily, the collection of the motifs that are found redundantly among the homologous proteins (see more details in [41,42]).

#### Confidence score calculation

The confidence scores, consisting of the *match score* (*MS*, Equation 1) and *conservation score* (*CS*, Equation 2), were defined in order to quantitatively determine the reliability of the cassava protein annotation. The *match score* (*MS*) indicates the confidence of the annotation relying on the quality of the alignment; whereas the *conservation score* (*CS*) represents the conservation of the protein across the plant species under study. In the annotation system where the multiple templates comprised of the species *i* ∈{*Arabidopsis*, rice, maize, castor bean, potato} were employed to identify the function of a query sequence, the definitions of *MS* and *CS* are given as follows:

(1) $$\mathrm{MS}=\frac{\sum_{i}^{N_{m}}\left(H_{i}\times F_{i}\right)}{N_{m}},$$

(2) $$\mathrm{CS}=\frac{\sum_{i}^{N_{m}}\left(H_{i}\times F_{i}\right)}{N_{t}}.$$

*H* denotes the quality of the match between a pair of sequence alignments, for which 1 was given to the RBH sequences and 0.5 was assigned to the others. *F* represents the clarity of the function of the hit templates where clear annotation was given as 1 and 0.5 otherwise. *N*_*t*_ = {5} and *N*_*m*_ = {1, …, 5} respectively represent a number of templates employed in this study and a number of templates from which the function of a query sequence was able to be identified. Both confidence scores range from 0 of no confidence to 1 of the highest confidence. An example of the confidence score calculation is provided in the Additional file 10.

### Pathway visualization and transcriptome data integration

#### Pathway visualization

The starch biosynthesis pathway of cassava was visualized on two platforms that deliberately convey different information. The descriptive map of the pathway that incorporates all information about the protein annotation and pathway reconstruction was drawn using the SmartDraw® 2012 (Figures 2, 3, 4). The interactive map of the pathway was developed using the Visualization and Analysis of Networks containing Experimental Data (VANTED v2.01) free tool (Additional file 2: Figure S1; Additional file 3: Figure S2; Additional file 4: Figure S3) [43,44], allowing further integration of omics data like transciptome data into the reconstructed metabolic pathway.

#### Transcriptome data integration

The microarray gene expression data in different types of cassava roots [7] were selected to exemplify the integration of the omics data into the biochemical pathways. To incorporate the gene expression data to the metabolic pathway, the cassava gene sequences comprised of the starch biosynthesis pathway were aligned against the probe library of the microarray experiment. The probes that matched well with the sequence of the gene of interest (E-value ≤ 1e-10 and identity percentage ≥ 95) were considered as the representatives of that gene and their expression could then be incorporated into the pathway. Note that only the significant genes whose expression passed the criteria of the two-fold change difference were selected to be integrated into the network.

### Availability of supporting data

The additional data files as well as the supplementary data mentioned in the manuscript are provided at the website of the Systems Biology and Bioinformatics research group at KMUTT (http://sbi.pdti.kmutt.ac.th/?page_id=33):

•The descriptive map of the carbon dioxide fixation process on the SmartDraw platform

•The descriptive map of the sucrose synthesis process on the SmartDraw platform

•The descriptive map of the starch synthesis process on the SmartDraw platform

•The pathway maps that include the results of the protein motif analysis

•The integrated map of the carbon dioxide fixation process on the VANTED platform

•The integrated map of the sucrose synthesis process on the VANTED platform

•The integrated map of the starch synthesis process on the VANTED platform

## Competing interests

The authors declare that they have no competing interests.

## Authors’ contributions

Conceived and designed experiment: TS, SK, AM, SC. Performed the computational analysis: OR, PC and WS. Analyzed the data and results: TS and SK. Discussed the results: TS, SK, SN and MS. Wrote the paper: TS, PC and WS. All authors read and approved the final manuscript.

## Supplementary Material

Additional file 1: Table S2. Comparison of the predicted starch-genes in cassava with Phytozome annotation. Click here for file

Additional file 2: Figure S1 The reconstructed pathway of the carbon dioxide fixation process in cassava presented on the VANTED platform. The boxes in front of each gene ID are the locations where the omics data are presented. Click here for file

Additional file 3: Figure S2 The reconstructed pathway of the sucrose synthesis process in cassava presented on the VANTED platform. The boxes in front of each gene ID are the locations where the omics data are presented. Click here for file

Additional file 4: Figure S3 The reconstructed pathway of the starch synthesis process in cassava presented on the VANTED platform. The boxes in front of each gene ID are the locations where the omics data are presented. Click here for file

Additional file 5 **The complete results of the protein motif analysis visualized in the interactive pathway maps as exemplified in Figure **5**.** Click here for file

Additional file 6: Figure S4 The reconstructed pathway of the carbon dioxide fixation process in cassava with the isozyme annotation presented on the SmartDraw platform. The number in the orange boxes denotes the EC number of the enzymes which is possibly a product of the genes below, denoted as the 12-digit ID. The colored dots beside each gene ID indicate the plant templates from which the genes were annotated: green – *Arabidopsis*, red – maize, pink – rice, violet – castor bean, and orange – potato. The background colors of the dots represent the matching quality of the sequence alignment: highest in yellow to lower in blue and the lowest in white. The following two columns describe the match (*MS*) and conservation (*CS*) scores, respectively. The box marks the reactions that are relevant to the C4-photosynthesis pathway found in a typical C4-plant. Click here for file

Additional file 7: Figure S5 The reconstructed pathway of the sucrose synthesis process in cassava with the isozyme annotation presented on the SmartDraw platform. The number in the orange boxes denotes the EC number of the enzymes which is possibly a product of the genes below, denoted as the 12-digit ID. The colored dots beside each gene ID indicate the plant templates from which the genes were annotated: green – *Arabidopsis*, red – maize, pink – rice, violet – castor bean, and orange – potato. The background colors of the dots represent the matching quality of the sequence alignment: highest in yellow to lower in blue and the lowest in white. The following two columns describe the match (*MS*) and conservation (*CS*) scores, respectively. Click here for file

Additional file 8: Figure S6 The reconstructed pathway of the starch synthesis process in cassava with the isozyme annotation presented on the SmartDraw platform. The number in the orange boxes denotes the EC number of the enzymes which is possibly a product of the genes below, denoted as the 12-digit ID. The colored dots beside each gene ID indicate the plant templates from which the genes were annotated: green – *Arabidopsis*, red – maize, pink – rice, violet – castor bean, and orange – potato. The background colors of the dots represent the matching quality of the sequence alignment: highest in yellow to lower in blue and the lowest in white. The following two columns describe the match (*MS*) and conservation (*CS*) scores, respectively. Click here for file

Additional file 9: Table S1 The existence of the annotated genes in cassava based on the Genbank database. The available data of cassava in the Genbank database were used to support the existence of the genes comprised of the reconstructed starch biosynthesis pathway. All annotated gene sequences were aligned against the sequences in the database using the following criteria for identification: E-value ≤ 10^-10^ and percent identity ≥ 90. Click here for file

Additional file 10 Supplementary information. Click here for file
