# Supplementary figures and images for: Starch biosynthesis in cassava: a genome-based pathway reconstruction and its exploitation in data integration
Source: BMC Syst Biol. 2013 Aug 10;7:75. doi: 10.1186/1752-0509-7-75 (PMC3847483; doi:10.1186/1752-0509-7-75)

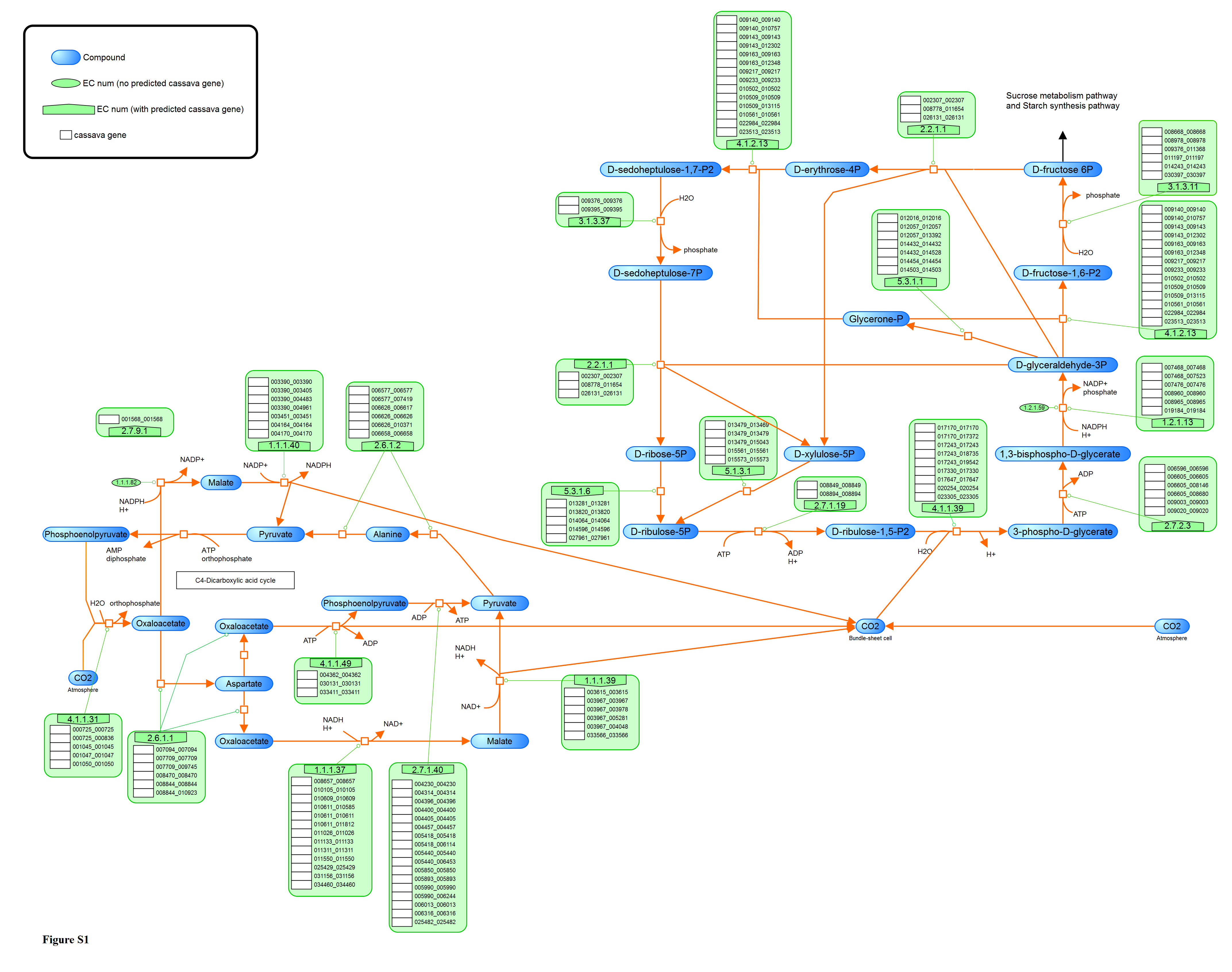

Supplement: Additional file 2: Figure S1 — The reconstructed pathway of the carbon dioxide fixation process in cassava presented on the VANTED platform. The boxes in front of each gene ID are the locations where the omics data are presented. [file 1752-0509-7-75-S2.png]

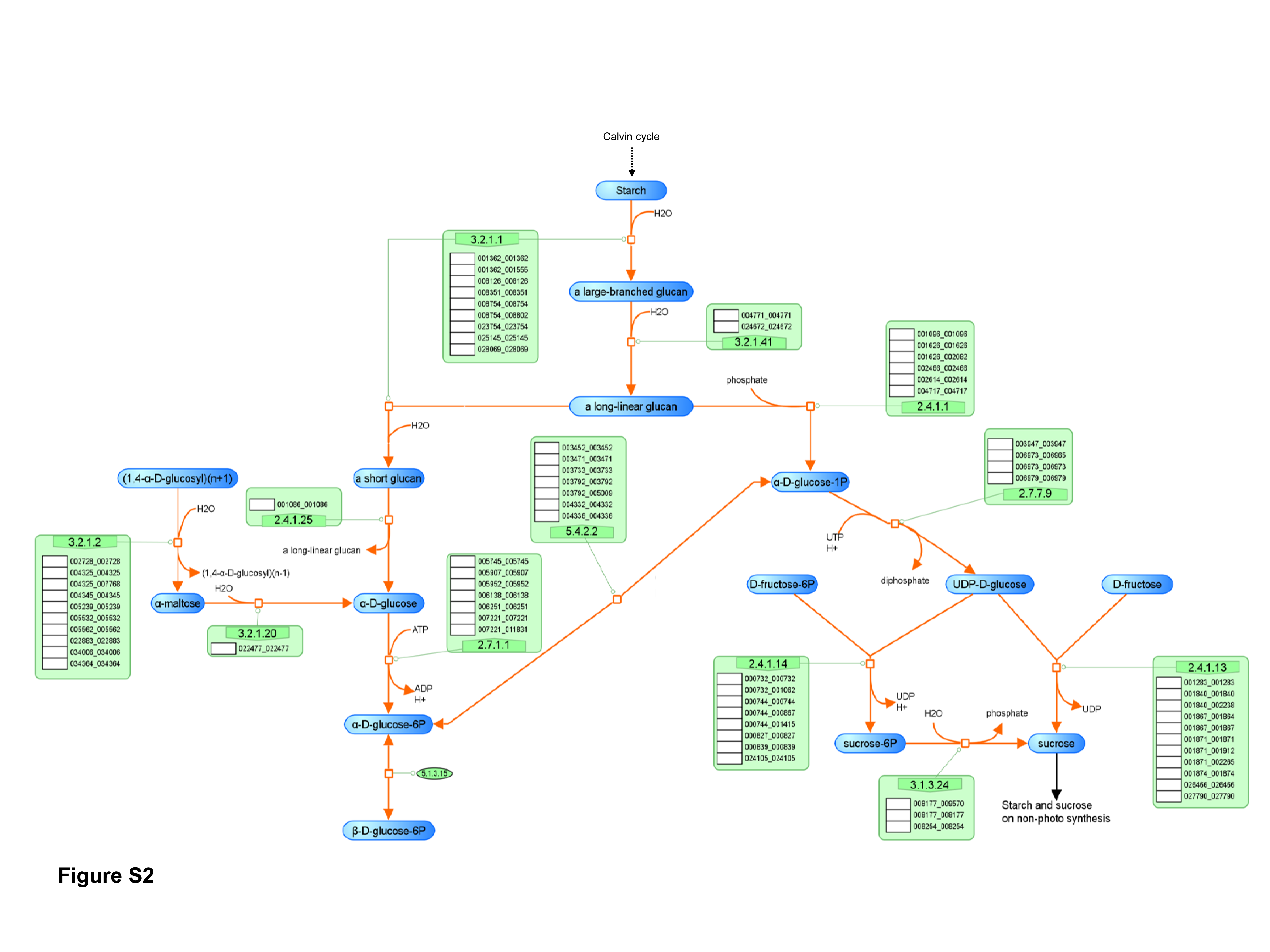

Supplement: Additional file 3: Figure S2 — The reconstructed pathway of the sucrose synthesis process in cassava presented on the VANTED platform. The boxes in front of each gene ID are the locations where the omics data are presented. [file 1752-0509-7-75-S3.tiff]

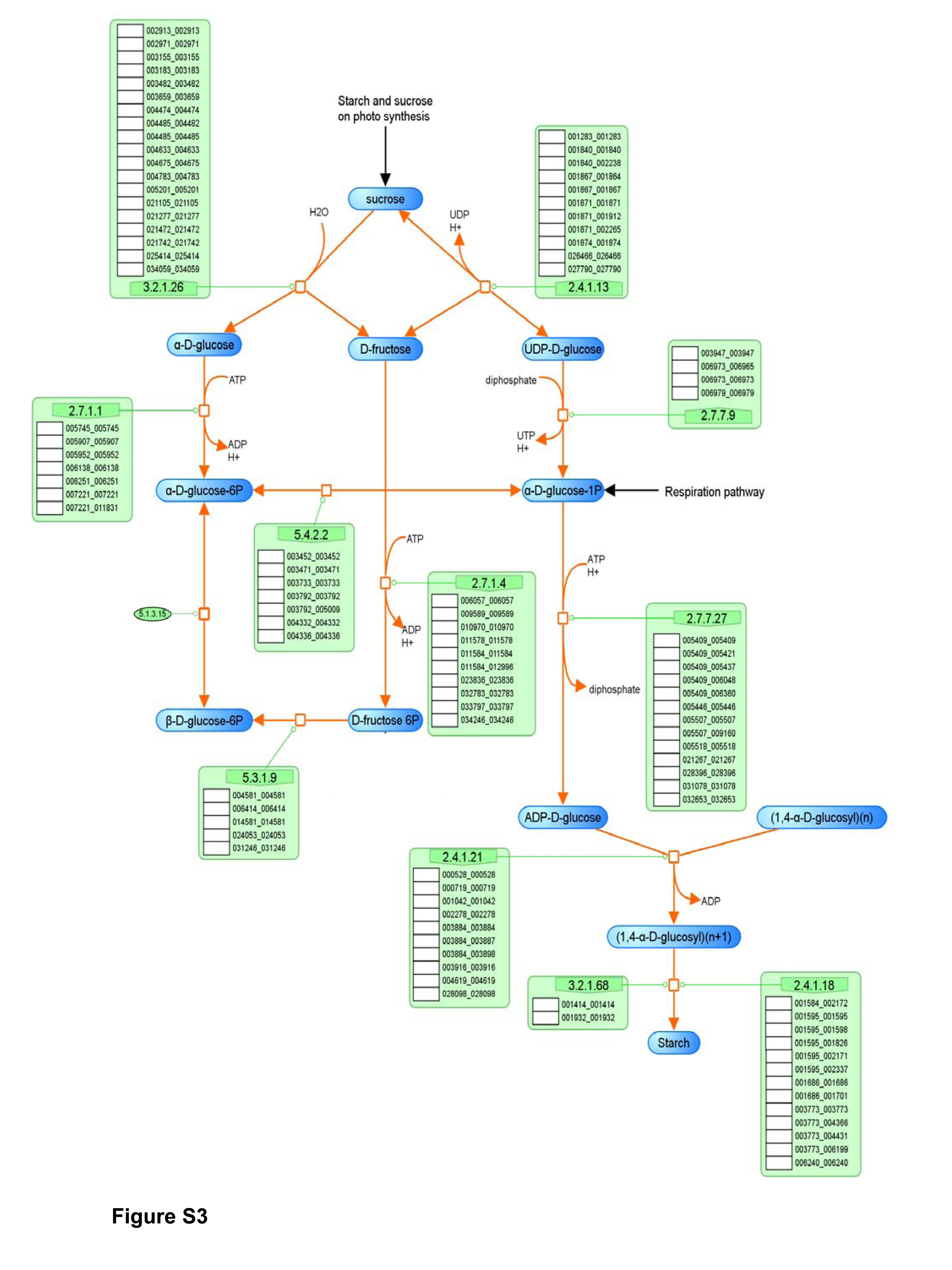

Supplement: Additional file 4: Figure S3 — The reconstructed pathway of the starch synthesis process in cassava presented on the VANTED platform. The boxes in front of each gene ID are the locations where the omics data are presented. [file 1752-0509-7-75-S4.tiff]

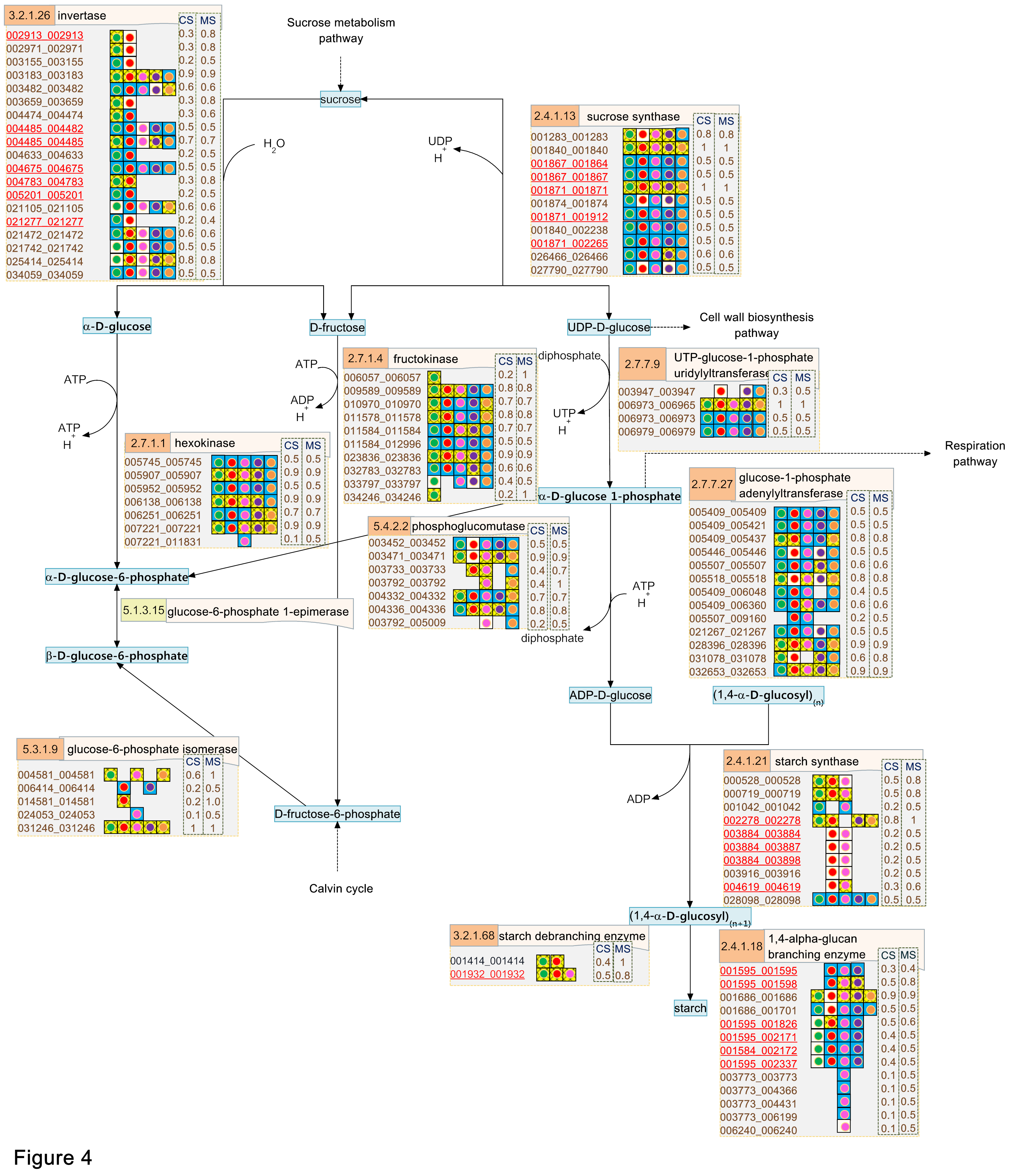

Supplement: Additional file 5 — The complete results of the protein motif analysis visualized in the interactive pathway maps as exemplified in Figure 5 . [file 1752-0509-7-75-S5.zip › SB pathway_HTML_22Feb13/starch synthesis.png]

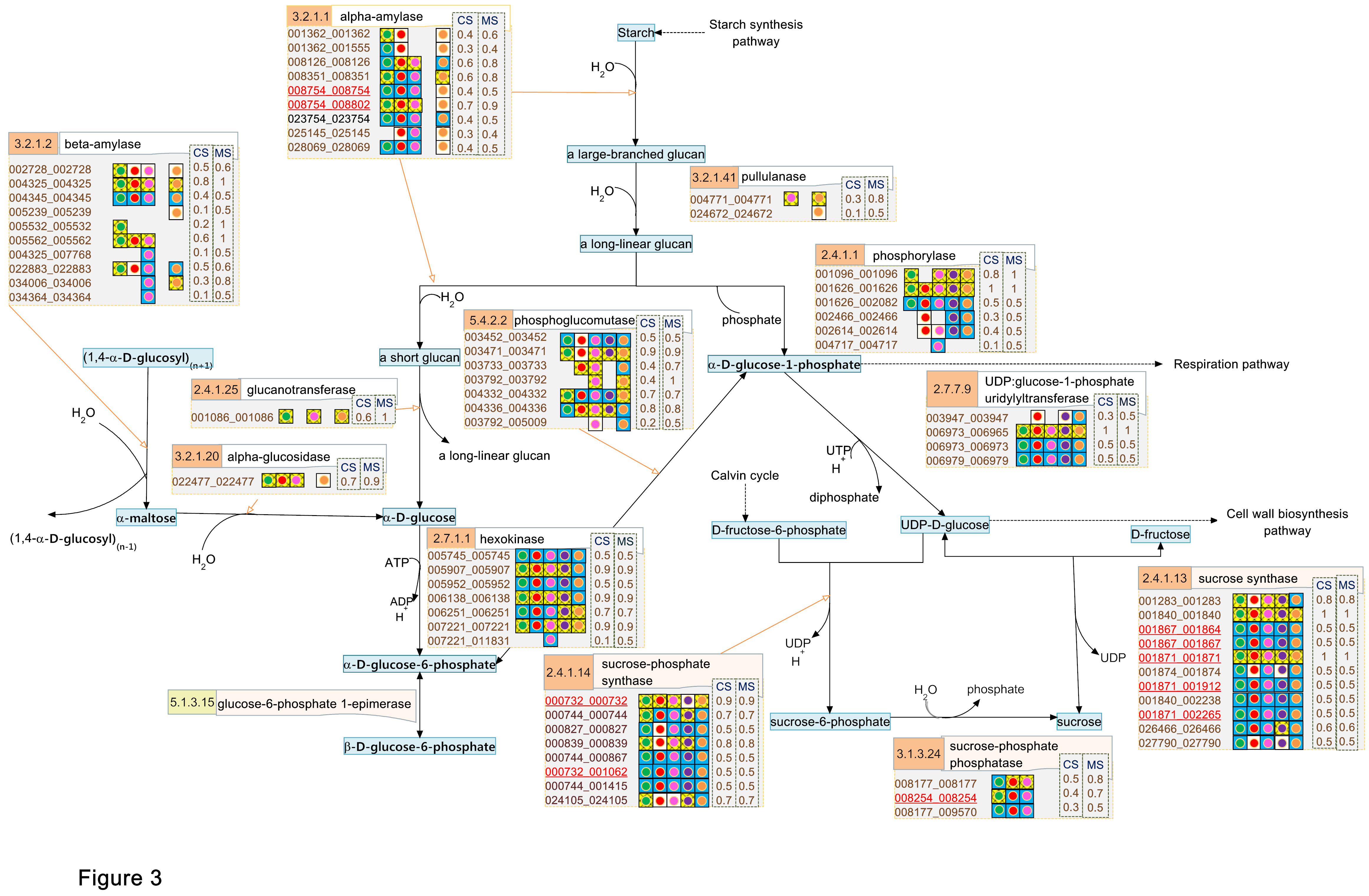

Supplement: Additional file 5 — The complete results of the protein motif analysis visualized in the interactive pathway maps as exemplified in Figure 5 . [file 1752-0509-7-75-S5.zip › SB pathway_HTML_22Feb13/sucrose synthesis.png]

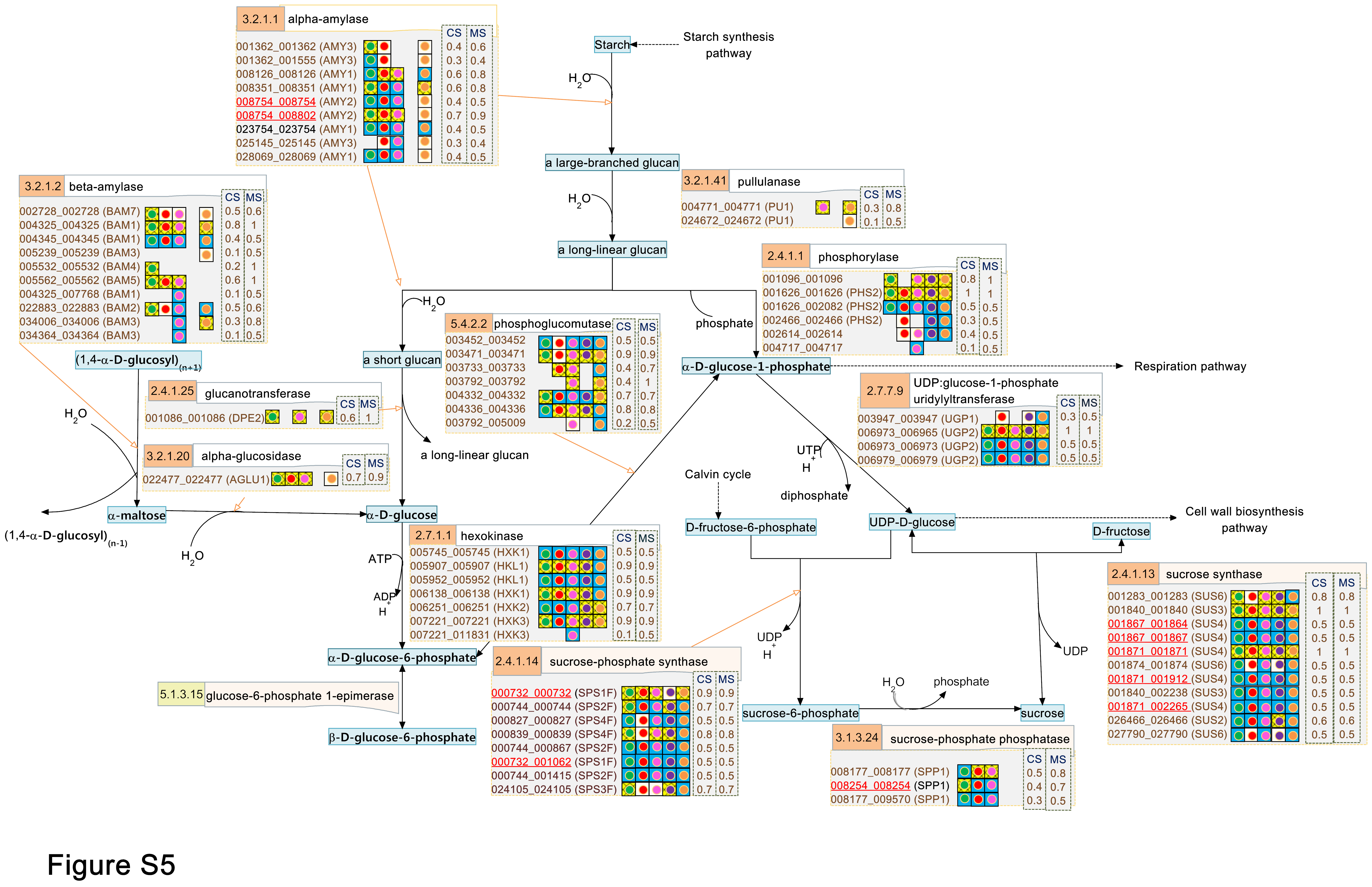

Supplement: Additional file 7: Figure S5 — The reconstructed pathway of the sucrose synthesis process in cassava with the isozyme annotation presented on the SmartDraw platform. The number in the orange boxes denotes the EC number of the enzymes which is possibly a product of the genes below, denoted as the 12-digit ID. The colored dots beside each gene ID indicate the plant templates from which the genes were annotated: green – Arabidopsis, red – maize, pink – rice, violet – castor bean, and orange – potato. The background colors of the dots represent the matching quality of the sequence alignment: highest in yellow to lower in blue and the lowest in white. The following two columns describe the match (MS) and conservation (CS) scores, respectively. [file 1752-0509-7-75-S7.tiff]

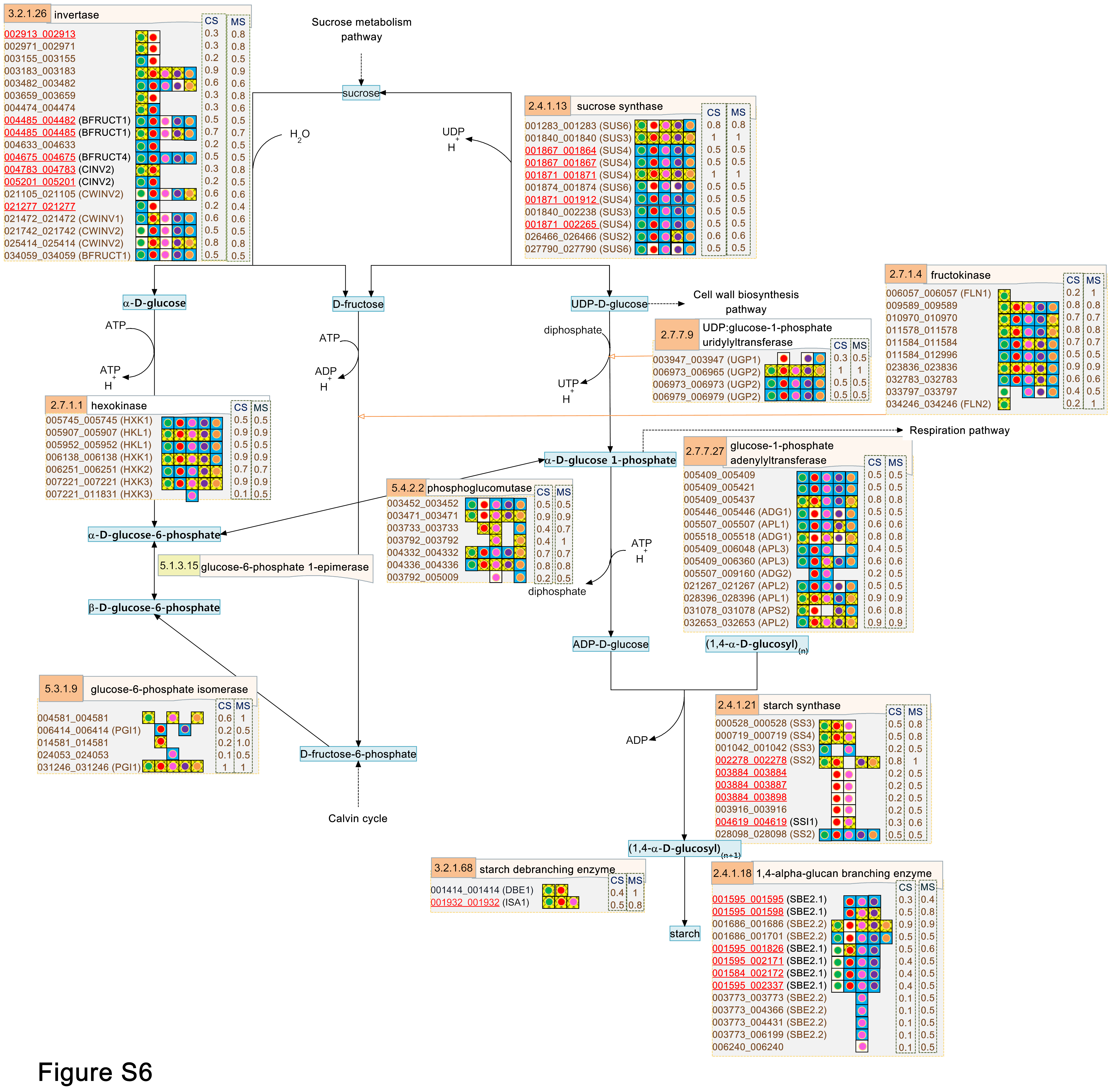

Supplement: Additional file 8: Figure S6 — The reconstructed pathway of the starch synthesis process in cassava with the isozyme annotation presented on the SmartDraw platform. The number in the orange boxes denotes the EC number of the enzymes which is possibly a product of the genes below, denoted as the 12-digit ID. The colored dots beside each gene ID indicate the plant templates from which the genes were annotated: green – Arabidopsis, red – maize, pink – rice, violet – castor bean, and orange – potato. The background colors of the dots represent the matching quality of the sequence alignment: highest in yellow to lower in blue and the lowest in white. The following two columns describe the match (MS) and conservation (CS) scores, respectively. [file 1752-0509-7-75-S8.tiff]
