# Supplementary material for: Starch biosynthesis in cassava: a genome-based pathway reconstruction and its exploitation in data integration
Source: BMC Syst Biol. 2013 Aug 10;7:75. doi: 10.1186/1752-0509-7-75 (PMC3847483; doi:10.1186/1752-0509-7-75)
Supplement: Additional file 5 — The complete results of the protein motif analysis visualized in the interactive pathway maps as exemplified in Figure 5 . [file 1752-0509-7-75-S5.zip › SB pathway_HTML_22Feb13/Calvin.html]

Calvin cycle


| 1.1.1.40 | cd05312 (cl09931) | cl02855 | cl09155 |
| 003390\_003405 |  |  |  |
| 003390\_004961 |  |  |  |
| 003390\_003390 |  |  |  |
| 003390\_004483 |  |  |  |
| 003451\_003451 |  |  |  |
| 004164\_004164 |  |  |  |
| 004170\_004170 |  |  |  |
| 004230\_004230 |  |  |  |
| 004314\_004314 |  |  |  |

|  |  |  |  |  |  |  |  |  |  |  |  |  |  |  |
| --- | --- | --- | --- | --- | --- | --- | --- | --- | --- | --- | --- | --- | --- | --- |
| | 2.6.1.2 | cl00321 (cd00609) | | 006577\_007419 |  | | 006577\_006577 |  | | 006626\_006617 |  | | 006626\_010371 |  | | 006626\_006626 |  | | 006658\_006658 |  | |

| 2.7.9.1 | cl09155 | cl08318 | cl01586 |
| 001568\_001568 |  |  |  |

| 4.1.31 | cl14656 |
| 000725\_000836 |  |
| 000725\_000725 |  |
| 001045\_001045 |  |
| 001047\_001047 |  |
| 001050\_001050 |  |

| 2.6.1.1 | cd00609 (cd00609) |
| 007094\_007094 |  |
| 007709\_007709 |  |
| 007709\_009745 |  |
| 008470\_008470 |  |
| 008844\_010923 |  |
| 008844\_008844 |  |

| 1.1.1.37 | cl09931 (cd01337) | cl09931 (cd01336) |
| 008657\_008657 |  |  |
| 010105\_010105 |  |  |
| 010609\_010609 |  |  |
| 010611\_010585 |  |  |
| 010611\_010611 |  |  |
| 010611\_011812 |  |  |
| 011026\_011026 |  |  |
| 011133\_011133 |  |  |
| 011550\_011550 |  |  |
| 025429\_025429 |  |  |
| 031156\_031156 |  |  |
| 034460\_034460 |  |  |

| 1.1.1.39 | cl09931 (cd05312) | cl02855 |
| 003615\_003615 |  |  |
| 003967\_003967 |  |  |
| 003967\_003978 |  |  |
| 003967\_005281 |  |  |
| 003967\_004048 |  |  |
| 033566\_033566 |  |  |

| 2.7.1.40 | cl09155 (cd00288) |
| 004396\_004396 |  |
| 004400\_004400 |  |
| 004405\_004405 |  |
| 004457\_004457 |  |
| 005418\_006114 |  |
| 005418\_005418 |  |
| 005893\_005893 |  |
| 005990\_006244 |  |
| 005440\_006453 |  |
| 005440\_005440 |  |
| 005990\_005990 |  |
| 006013\_006013 |  |
| 006316\_006316 |  |
| 025482\_025482 |  |

| 4.1.1.49 | cl00270 (cd00484) |
| 004362\_004362 |  |
| 030131\_030131 |  |
| 033411\_033411 |  |

| 4.1.2.13 | cl09108 (cd00948) |
| 009140\_010757 |  |
| 009140\_009140 |  |
| 009143\_012302 |  |
| 009143\_009143 |  |
| 009163\_009163 |  |
| 009163\_012348 |  |
| 009217\_009217 |  |
| 009233\_009233 |  |
| 010502\_010502 |  |
| 010509\_013115 |  |
| 010509\_010509 |  |
| 010561\_010561 |  |
| 022984\_022984 |  |
| 023513\_023513 |  |

| 3.1.3.37 | cl00289 (cd00354) |
| 009376\_009376 |  |
| 009395\_009395 |  |

| 2.2.1.1 | cl01629 (cd02012) | cl11410 (cd07033) | cl11410 (cd07036) | cl08363 |
| 002307\_002307 |  |  |  |  |
| 008778\_011654 |  |  |  |  |
| 026131\_026131 |  |  |  |  |

| 5.3.1.6 | cl00339 (cd01398) |
| 013281\_013281 |  |
| 013820\_013820 |  |
| 014064\_014064 |  |
| 014596\_014596 |  |
| 027961\_027961 |  |

| 2.7.1.19 | cl09099 (cd02026) |
| 008849\_008849 |  |
| 008894\_008894 |  |

| 5.1.3.1 | cl09108 (cd00429) |
| 013479\_013469 |  |
| 013479\_015043 |  |
| 013479\_013479 |  |
| 015561\_015561 |  |
| 015573\_015573 |  |

| 4.1.1.39 | cl01843 (cd03527) | cl13735 | cl08232 |
| 017170\_017170 |  |  |  |
| 017170\_017372 |  |  |  |
| 017243\_019542 |  |  |  |
| 017243\_017243 |  |  |  |
| 017243\_018735 |  |  |  |
| 017330\_017330 |  |  |  |
| 017647\_017647 |  |  |  |
| 020254\_020254 |  |  |  |
| 023305\_023305 |  |  |  |

| 2.7.2.3 | cl00198 (cd00318) |
| 006596\_006596 |  |
| 006605\_008680 |  |
| 006605\_006605 |  |
| 006605\_008146 |  |
| 009003\_009003 |  |
| 009020\_009020 |  |

| 1.2.1.13 | cl15856 | cl09931 | cl14670 |
| 007468\_007523 |  |  |  |
| 007468\_007468 |  |  |  |
| 007476\_007476 |  |  |  |
| 008960\_008960 |  |  |  |
| 008965\_008965 |  |  |  |
| 019184\_019184 |  |  |  |

| 3.1.3.11 | cl00289 (cd00354) |
| 008668\_008668 |  |
| 008978\_008978 |  |
| 009376\_011368 |  |
| 009376\_009376 |  |
| 009395\_009395 |  |
| 011197\_011197 |  |
| 014243\_014243 |  |
| 030397\_030397 |  |
