# Supplementary material for: Starch biosynthesis in cassava: a genome-based pathway reconstruction and its exploitation in data integration
Source: BMC Syst Biol. 2013 Aug 10;7:75. doi: 10.1186/1752-0509-7-75 (PMC3847483; doi:10.1186/1752-0509-7-75)
Supplement: Additional file 5 — The complete results of the protein motif analysis visualized in the interactive pathway maps as exemplified in Figure 5 . [file 1752-0509-7-75-S5.zip › SB pathway_HTML_22Feb13/Starch synthesis.html]

Starch synthesis
 

| 3.2.1.26 | cl01801 | cl14647  (cd08996) | cl13304 | cl07030 |
| 002913\_002913 |  |  |  |  |
| 002971\_002971 |  |  |  |  |
| 003155\_003155 |  |  |  |  |
| 003183\_003183 |  |  |  |  |
| 003482\_003482 |  |  |  |  |
| 003659\_003659 |  |  |  |  |
| 004474\_004474 |  |  |  |  |
| 004485\_004482 |  |  |  |  |
| 004485\_004485 |  |  |  |  |
| 004633\_004633 |  |  |  |  |
| 004675\_004675 |  |  |  |  |
| 004783\_004783 |  |  |  |  |
| 005201\_005201 |  |  |  |  |
| 021105\_021105 |  |  |  |  |
| 021277\_021277 |  |  |  |  |
| 021472\_021472 |  |  |  |  |
| 021742\_021742 |  |  |  |  |
| 025414\_025414 |  |  |  |  |
| 034059\_034059 |  |  |  |  |

| 5.3.1.9 | cl00389 (cd05016) | cl00357 |
| 004581\_004581 |  |  |
| 006414\_006414 |  |  |
| 014581\_014581 |  |  |
| 024053\_024053 |  |  |
| 031246\_031246 |  |  |

| 2.7.1.4 | cl00192 (cd01167) |
| 006057\_006057 |  |
| 009589\_009589 |  |
| 010970\_010970 |  |
| 011578\_011578 |  |
| 011584\_012996 |  |
| 023836\_023836 |  |
| 032783\_032783 |  |
| 033797\_033797 |  |
| 034246\_034246 |  |

| 2.7.7.27 | cl11394 (cd02508) | cl00160 (cd04651) | cl12403 |
| 005409\_005421 |  |  |  |
| 005409\_005437 |  |  |  |
| 005446\_005446 |  |  |  |
| 005507\_005507 |  |  |  |
| 005518\_005518 |  |  |  |
| 005409\_006048 |  |  |  |
| 005409\_006360 |  |  |  |
| 005507\_009160 |  |  |  |
| 021267\_021267 |  |  |  |
| 028396\_028396 |  |  |  |
| 031078\_031078 |  |  |  |
| 032653\_032653 |  |  |  |

| 2.4.1.21 | cl10013 (cd03791) | cl04060 | cl10824 |
| 000528\_000528 |  |  |  |
| 000719\_000719 |  |  |  |
| 001042\_001042 |  |  |  |
| 002278\_002278 |  |  |  |
| 003884\_003884 |  |  |  |
| 003884\_003887 |  |  |  |
| 003884\_003898 |  |  |  |
| 003916\_003916 |  |  |  |
| 004619\_004619 |  |  |  |
| 028098\_028098 |  |  |  |

| 2.4.1.18 | cl09101 (cd02854) | cl02706 | cl07893 |
| 001595\_001595 |  |  |  |
| 001595\_001598 |  |  |  |
| 001686\_001686 |  |  |  |
| 001686\_001701 |  |  |  |
| 001595\_001826 |  |  |  |
| 001595\_002171 |  |  |  |
| 001584\_002172 |  |  |  |
| 001595\_002337 |  |  |  |
| 003773\_003773 |  |  |  |
| 003773\_004366 |  |  |  |
| 003773\_004431 |  |  |  |
| 003773\_006199 |  |  |  |
| 006240\_006240 |  |  |  |

| 3.2.1.68 | cl09101 (cd02852) | cl07893 |
| 001414\_001414 |  |  |
| 001932\_001932 |  |  |

|  |  |  |
| --- | --- | --- |
|  |  |  |
| ................................................. | 2.7.1.1 | cl08262 | cl08402 | | 005745\_005745 |  |  | | 005907\_005907 |  |  | | 005952\_005952 |  |  | | 006138\_006138 |  |  | | 006251\_006251 |  |  | | 007221\_007221 |  |  | | 007221\_011831 |  |  | ..... |  | | 2.4.1.13 | cl10013 (cd03800) | | 001283\_001283 |  | | 001840\_001840 |  | | 001867\_001864 |  | | 001867\_001867 |  | | 001871\_001871 |  | | 001874\_001874 |  | | 001871\_001912 |  | | 001840\_002238 |  | | 001871\_002265 |  | | 026466\_026466 |  | | 027790\_027790 |  | |
|  |  |  |

| 2.7.7.9 | cl11394 (cd00897) | cl00315 (cd01425) |
| 003947\_003947 |  |  |
| 006973\_006965 |  |  |
| 006973\_006973 |  |  |
| 006979\_006979 |  |  |

| 5.4.2.2 | cl03757 (cd03085) |
| 003452\_003452 |  |
| 003471\_003471 |  |
| 003733\_003733 |  |
| 003792\_003792 |  |
| 004332\_004332 |  |
| 004336\_004336 |  |
