# Supplementary material for: Starch biosynthesis in cassava: a genome-based pathway reconstruction and its exploitation in data integration
Source: BMC Syst Biol. 2013 Aug 10;7:75. doi: 10.1186/1752-0509-7-75 (PMC3847483; doi:10.1186/1752-0509-7-75)
Supplement: Additional file 5 — The complete results of the protein motif analysis visualized in the interactive pathway maps as exemplified in Figure 5 . [file 1752-0509-7-75-S5.zip › SB pathway_HTML_22Feb13/Sucrose synthesis.html]

Sucrose synthesis
 

| 2.4.1.1 | cl10013  (cd04300) |
| 001096\_001096 |  |
| 001626\_001626 |  |
| 001626\_002082 |  |
| 002466\_002466 |  |
| 002614\_002614 |  |

| 2.4.1.14 | cd03800 | cl10013 |
| 000732\_000732 |  |  |
| 000744\_000744 |  |  |
| 000827\_000827 |  |  |
| 000839\_000839 |  |  |
| 000744\_000867 |  |  |
| 000732\_001062 |  |  |
| 000744\_001415 |  |  |
| 024105\_024105 |  |  |

| 3.1.3.24 | cl07189 |
| 008177\_008177 |  |
| 008254\_008254 |  |
| 008177\_009570 |  |

| 2.4.1.13 | cl10013 (cd03800) |
| 001283\_001283 |  |
| 001840\_001840 |  |
| 001867\_001864 |  |
| 001867\_001867 |  |
| 001871\_001871 |  |
| 001874\_001874 |  |
| 001871\_001912 |  |
| 001840\_002238 |  |
| 001871\_002265 |  |
| 026466\_026466 |  |
| 027790\_027790 |  |

|  |  |  |
| --- | --- | --- |
|  |  |  |
| ................................................ | | 2.7.1.1 | cl08262 | cl08402 | | 005745\_005745 |  |  | | 005907\_005907 |  |  | | 005952\_005952 |  |  | | 006138\_006138 |  |  | | 006251\_006251 |  |  | | 007221\_007221 |  |  | | 007221\_011831 |  |  |  | 3.2.1.2 | cl03185 | cl05316 | | 002728\_002728 |  |  | | 004325\_004325 |  |  | | 004345\_004345 |  |  | | 005239\_005239 |  |  | | 005532\_005532 |  |  | | 005562\_005562 |  |  | | 004325\_007768 |  |  | | 022883\_022883 |  |  | | 034006\_034006 |  |  | | 034364\_034364 |  |  |  | 3.2.1.20 | cl11402 (cd06602) | | 022477\_022477 |  |  | 5.4.2.2 | cl03757 (cd03085) | | 003452\_003452 |  | | 003471\_003471 |  | | 003733\_003733 |  | | 003792\_003792 |  | | 004332\_004332 |  | | 004336\_004336 |  |  | 3.2.1.41 | cl09101 (cd02860) | cl13318 | cl07893 | | 004771\_004771 |  |  |  | | 024672\_024672 |  |  |  |  | 2.7.7.9 | cl11394 (cd00897) | cl00315 (cd01425) | | 003947\_003947 |  |  | | 006973\_006965 |  |  | | 006973\_006973 |  |  | | 006979\_006979 |  |  | |  |
|  | | 2.4.1.25 | cl08281 (cd05815) | cl08281 | cl00711 | | 001086\_001086 |  |  |  | |  |

| 3.2.1.1 | cl06726 | cl07893 |
| 001362\_001362 |  |  |
| 001362\_001555 |  |  |
| 008126\_008126 |  |  |
| 008351\_008351 |  |  |
| 008754\_008754 |  |  |
| 008754\_008802 |  |  |
| 023754\_023754 |  |  |
| 025145\_025145 |  |  |
| 028069\_028069 |  |  |
