# Supplementary material for: Starch biosynthesis in cassava: a genome-based pathway reconstruction and its exploitation in data integration
Source: BMC Syst Biol. 2013 Aug 10;7:75. doi: 10.1186/1752-0509-7-75 (PMC3847483; doi:10.1186/1752-0509-7-75)
Supplement: Additional file 9: Table S1 — The existence of the annotated genes in cassava based on the Genbank database. The available data of cassava in the Genbank database were used to support the existence of the genes comprised of the reconstructed starch biosynthesis pathway. All annotated gene sequences were aligned against the sequences in the database using the following criteria for identification: E-value ≤ 10-10 and percent identity ≥ 90. [file 1752-0509-7-75-S9.docx]

**Table S1** The existence of the annotated genes in cassava based on Genbank database

The available data of cassava in the Genbank database were used to support the existence of the genes comprised of the reconstructed starch biosynthesis pathway. All annotated gene sequences were aligned against the sequences in the database using the following criteria for identification: E-value ≤ 10-10 and percent identity ≥ 90.

| Experimentally identified genes in GenBank database | GenBank function | The corresponding genes identified in this work | Identity (Percent) | E-value |
| --- | --- | --- | --- | --- |
| gi\|157169067\|gb\|ABV25893.1\| | Starch synthase I [Manihot esculenta] | 004619_004619 | 99.45 | 0 |
| gi\|157169069\|gb\|ABV25894.1\| | Starch synthase II [Manihot esculenta] | 002278_002278 | 100 | 0 |
| gi\|1771261\|emb\|CAA54308.1\| | 1,4-alpha-glucan branching enzyme [Manihot esculenta] | 001595_002171  001584_002172  001595_001595  001595_001598  001595_002337  001595_001826 | 98.82  98.82  98.47  98.47  97.30  94.37 | 0  0  0  0  0  0 |
| gi\|218546884\|sp\|B1NWF8.1\|RBL_MANES | RuBisCO large subunit | 023305_023305  020254_020254 | 97.44  96.63 | 3E-34  1E-49 |
| gi\|2833388\|sp\|Q43784.1\|SSG1_MANES | Granule-bound starch synthase I; GBSSI | 003884_003884  003884_003887  003884_003898 | 99.34  99.34  99.34 | 0  0  0 |
| gi\|291293217\|gb\|ADD92152.1\| | Sucrose phosphate synthase [Manihot esculenta] | 000732_000732  000732_001062 | 100  100 | 1E-121  1E-121 |
| gi\|291293219\|gb\|ADD92153.1\| | Sucrose phosphatase [Manihot esculenta] | 008254_008254 | 99.34 | 1E-89 |
| gi\|295821001\|gb\|ADG36408.1\| | Isoamylase [Manihot esculenta] | 001932_001932 | 99.61 | 8E-154 |
| gi\|343175396\|gb\|AEM00023.1\| | Vacuolar acid invertase [Manihot esculenta] | 004675_004675 | 96.96 | 0 |
| gi\|347949208\|gb\|AEP31948.1\| | Neutral/alkaline invertase [Manihot esculenta] | 002913_002913 | 99.71 | 0 |

**Table S1** Summary of cassava genes experimentally identified and submitted in GenBank (cont’d)

| Experimentally identified genes in GenBank database | GenBank function | The corresponding genes identified in this work | Identity (Percent) | E-value |
| --- | --- | --- | --- | --- |
| gi\|375300670\|gb\|AFA46812.1\| | Cell wall invertase [Manihot esculenta] | 004485_004485  004485_004482 | 99.31  99.13 | 0  0 |
| gi\|375300672\|gb\|AFA46813.1\| | Neutral/alkaline invertase [Manihot esculenta] | 021277_021277 | 99.12 | 0 |
| gi\|3914608\|sp\|Q42915.1\|RBS_MANES | RuBisCO small subunit | 017243_017243 | 100 | 1E-108 |
| gi\|56122688\|gb\|AAV74407.1\| | Chloroplast latex aldolase-like protein [Manihot esculenta] | 009143_009143  009143_012302 | 99.68  99.67 | 0  4E-180 |
| gi\|6272548\|gb\|AAF06098.1\| | Ribulose 1,5-bisphosphate carboxylase small chain precursor [Manihot esculenta] | 017170_017170  017170_017372 | 100  99.32 | 2E-109  2E-85 |
| gi\|68053506\|gb\|AAY85174.1\| | Alpha-amylase 2 [Manihot esculenta] | 008754_008802  008754_008754 | 96.09  95.85 | 0  0 |
| gi\|74476783\|gb\|ABA08442.1\| | Neutral/alkaline invertase [Manihot esculenta] | 005201_005201  004783_004783 | 99.81  96.59 | 0  0 |
| gi\|90655945\|gb\|ABD96570.1\| | Sucrose synthase [Manihot esculenta] | 001867_001864  001867_001867  001871_001871  001871_002265  001871_001912 | 99.63  99.63  96.53  96.14  95.66 | 0  0  0  0  0 |
