# Supplementary material for: Starch biosynthesis in cassava: a genome-based pathway reconstruction and its exploitation in data integration
Source: BMC Syst Biol. 2013 Aug 10;7:75. doi: 10.1186/1752-0509-7-75 (PMC3847483; doi:10.1186/1752-0509-7-75)
Supplement: Additional file 10 — Supplementary information. [file 1752-0509-7-75-S10.docx]

**Supplementary information**

**Example of the confidence score calculation**

To illustrate the practical use of our developed confidence scores to quantify the reliability of an annotated sequence, the example of the calculation is provided. For a cassava protein sequence that was annotated by being the reciprocal best hit (RBH) sequence of its orthologs in the two template plants, *i.e.* Arabidopsis and castor bean, and by being the non-RBH sequence of its putative ortholog in potato, the confidence scores can be computed as follows:

According to the given example,

*N_t_* = 5

*N_m_* = 3 (The sequence was annotated by its rothologs in the three templat plants.)

*i* = {Arabidopsis, castor bean, potato}

*H_Arabidopsis_* and *H_castor bean_* = 1 (RBH sequence)

*H_potato_* = 0.5 (non-RBH sequence)

*F_Arabidopsis_,* *F_castor bean_*  and *F_potato_* = 1 (the template sequence of the reciprocal has a clear annotated function in its genome)

- In case of *match score* (*MS*):

$$MS=\frac{\sum_{i}^{N_{m}} \left(H_{i}\times F_{i}\right)}{N_{m}}$$

$$MS=\frac{(H_{Arabidopsis}\times F_{Arabidopsis})+(H_{castorbean}\times F_{castorbean})+(H_{patato}\times F_{potato})}{3}$$

$$MS=\frac{(1\times1)+(1\times1)+(0.5\times1)}{3}$$

$$MS=\frac{(1\times1)+(1\times1)+(0.5\times1)}{3}$$

$$MS=0.83$$

- In case of *conservation score* (*CS*):

$$CS=\frac{\sum_{i}^{N_{m}} \left(H_{i}\times F_{i}\right)}{N_{t}}$$

$$CS=\frac{(H_{Arabidopsis}\times F_{Arabidopsis})+(H_{castorbean}\times F_{castorbean})+(H_{patato}\times F_{potato})}{5}$$

$$CS=\frac{(1\times1)+(1\times1)+(0.5\times1)}{5}$$

$$CS=\frac{(1\times1)+(1\times1)+(0.5\times1)}{5}$$

$$CS=0.5$$
